# Plant Salinity Tolerance Conferred by Arbuscular Mycorrhizal Fungi and Associated Mechanisms: A Meta-Analysis

**DOI:** 10.3389/fpls.2020.588550

**Published:** 2020-12-09

**Authors:** Khondoker M. G. Dastogeer, Mst Ishrat Zahan, Md. Tahjib-Ul-Arif, Mst Arjina Akter, Shin Okazaki

**Affiliations:** ^1^Graduate School of Agriculture, Tokyo University of Agriculture and Technology, Fuchu, Japan; ^2^Department of Plant Pathology, Bangladesh Agricultural University, Mymensingh, Bangladesh; ^3^Independent Researcher, Mymensingh, Bangladesh; ^4^Department of Biochemistry and Molecular Biology, Bangladesh Agricultural University, Mymensingh, Bangladesh

**Keywords:** AMF, antioxidant, standardized mean difference, plant physiology, photosynthesis, plant biomass, phylogenetic signal, effect size

## Abstract

Soil salinity often hinders plant productivity in both natural and agricultural settings. Arbuscular mycorrhizal fungal (AMF) symbionts can mediate plant stress responses by enhancing salinity tolerance, but less attention has been devoted to measuring these effects across plant-AMF studies. We performed a meta-analysis of published studies to determine how AMF symbionts influence plant responses under non-stressed vs. salt-stressed conditions. Compared to non-AMF plants, AMF plants had significantly higher shoot and root biomass (*p* < 0.0001) both under non-stressed conditions and in the presence of varying levels of NaCl salinity in soil, and the differences became more prominent as the salinity stress increased. Categorical analyses revealed that the accumulation of plant shoot and root biomass was influenced by various factors, such as the host life cycle and lifestyle, the fungal group, and the duration of the AMF and salinity treatments. More specifically, the effect of *Funneliformis* on plant shoot biomass was more prominent as the salinity level increased. Additionally, under stress, AMF increased shoot biomass more on plants that are dicots, plants that have nodulation capacity and plants that use the C3 plant photosynthetic pathway. When plants experienced short-term stress (<2 weeks), the effect of AMF was not apparent, but under longer-term stress (>4 weeks), AMF had a distinct effect on the plant response. For the first time, we observed significant phylogenetic signals in plants and mycorrhizal species in terms of their shoot biomass response to moderate levels of salinity stress, i.e., closely related plants had more similar responses, and closely related mycorrhizal species had similar effects than distantly related species. In contrast, the root biomass accumulation trait was related to fungal phylogeny only under non-stressed conditions and not under stressed conditions. Additionally, the influence of AMF on plant biomass was found to be unrelated to plant phylogeny. In line with the greater biomass accumulation in AMF plants, AMF improved the water status, photosynthetic efficiency and uptake of Ca and K in plants irrespective of salinity stress. The uptake of N and P was higher in AMF plants, and as the salinity increased, the trend showed a decline but had a clear upturn as the salinity stress increased to a high level. The activities of malondialdehyde (MDA), peroxidase (POD), and superoxide dismutase (SOD) as well as the proline content changed due to AMF treatment under salinity stress. The accumulation of proline and catalase (CAT) was observed only when plants experienced moderate salinity stress, but peroxidase (POD) and superoxide dismutase (SOD) were significantly increased in AMF plants irrespective of salinity stress. Taken together, arbuscular mycorrhizal fungi influenced plant growth and physiology, and their effects were more notable when their host plants experienced salinity stress and were influenced by plant and fungal traits.

## Introduction

Salinity is a major environmental problem that limits agricultural productivity worldwide, especially in arid and semiarid regions (Munns and Gilliham, 2015). The deleterious effect of NaCl on plants is caused by both the reduced water availability as sodium accumulation reduces the soil water potential and the toxic effects of sodium and chlorine ions on plants. Reduced water and nutrient uptake lead to osmotic stress, ion toxicity, and nutrient imbalances, resulting in significant reductions in plant growth and crop production (Munns and Tester, 2008; Hanin et al., 2016). Soil salinity problems are projected to worsen in the coming years in many low-lying areas due to the changing climate (Zörb et al., 2019). Plants adapt physiologically and biochemically to mitigate the detrimental effects of salinity via ion homeostasis and compartmentalization, osmoprotectant and compatible solute biosynthesis, antioxidant enzyme activation, antioxidant compound synthesis, polyamine synthesis, nitric oxide (NO) generation, and hormone modulation (Gupta and Huang, 2014; Hernández, 2019; Van Zelm et al., 2020). Plant responses to stress have been studied extensively in the last few decades, and the role of plant-microbe interactions on plant stress responses has also been given attention in recent years. In particular, plant species are commonly associated with fungal symbionts such as mycorrhizal fungi and endophytes (within plants), which may influence their responses to environmental stimuli, including salinity stress.

The majority of terrestrial plants form mutualistic associations with arbuscular mycorrhizal fungi (AMF). Accumulating evidence suggests that AMF colonization in roots can help improve plant tolerance to salinity stress (Al-Karaki et al., 2001; Porcel et al., 2012; Begum et al., 2019; Evelin et al., 2019). AMF employ various mechanisms to mitigate plant salinity stress. For instance, AMF can augment nutrient uptake, increase water uptake, maintain osmotic balance, stimulate antioxidant activities to protect against damage by reactive oxygen species (ROS), increase the photosynthetic rate and regulate hormonal levels to abate the harmful effects of salts on plant growth and development (Evelin et al., 2009, 2019; Ruiz-Lozano et al., 2012; Augé et al., 2014; Khalloufi et al., 2017). Under salinity stress, AMF increase the uptake of plant nutrients such as P (phosphorus), N (nitrogen), K (potassium), Zn (zinc), and Cu (copper) and maintain ionic homeostasis (Marschner and Dell, 1994; Pang et al., 2007; Sheng et al., 2011). The accumulation of proline is another mechanism associated with AMF-mediated plant salinity tolerance. However, the role of AMF in proline accumulation in plants is not consistent: several studies reported a higher proline content, whereas others reported a lower proline content in AMF-colonized plants under stress (Evelin et al., 2012; Hashem et al., 2015; Frosi et al., 2016). An efficient reactive oxygen species (ROS) scavenging system is paramount for alleviating salinity stress in plants. AMF colonization boosts the production of antioxidant molecules and enhances the activities of enzymes to provide an improved oxidation scavenging system (Serbinova and Packer, 1994; Evelin and Kapoor, 2014). Under salinity stress, increased activities of catalase (CAT), peroxidase (POD), superoxide dismutase (SOD), and ascorbate peroxidase (APX) have been reported in AMF-colonized plants compared to non-colonized plants in many studies (Li et al., 2012; Pandey and Garg, 2017; Hashem et al., 2018). Salinity stress affects stomatal conductance, disrupts photosynthetic machinery and decreases the activity of photosynthetic pigments, all of which impede photosynthesis in plants (Giri and Mukerji, 2004; Murkute et al., 2006; Sheng et al., 2008; Chaves et al., 2009). AMF help plants maintain water status, increase stomatal conductance and enhance photosynthetic pigments to combat the effects of salts and increase photosynthesis for growth and development (Hidri et al., 2016; Chen et al., 2017). In the last several years, considerable progress has been made to understand these mechanisms. Studies have examined various mechanisms considering different plant-AMF combinations. For a better understanding of the comprehensive biochemical and physiological mechanisms across host AMF settings, we need to systematically examine these studies to determine the relative importance of each mechanism. The magnitude of the effect of AMF on plant salinity tolerance differs greatly among various studies (Evelin et al., 2009; Porcel et al., 2012; Chandrasekaran et al., 2019). These differences can be attributed to various factors, such as the level of salinity, types of hosts and mycorrhizal partners, environmental conditions, and their complex interactions. Understanding the contribution of these factors to AMF-plant symbiosis is important to elucidate the mechanism of AMF-mediated plant salinity tolerance. It is a complex task to infer general findings from individual studies. Therefore, to determine a central tendency, identify different patterns of AMF influences on plants under stress and compare them with those under control conditions, it is paramount to integrate results from multiple studies to determine whether general factors can be identified. To this end, we conducted a meta-analysis to measure the overall magnitude and direction of the summary effect size of AMF symbiosis on important plant characteristics associated with stress tolerance mechanisms.

A meta-analysis is a mathematical approach that combines data from different studies using weighted statistical methods to calculate a mean effect size for the treatment across a range of studies (Rosenberg et al., 2000). Meta-analysis helps us to understand the results of a study in the context of all other comparable studies to evaluate whether the effect of a particular treatment is coherent across studies, whether it is noticeably different across studies, and which element might be responsible for this disparity (Borenstein et al., 2009). Categorical variables or “moderators” are often included in meta-analyses to determine how various features modulate the treatment effect of interest. Meta-analyses have been used broadly in various disciplines and have become increasingly common in plant ecology and evolutionary biology (Lau et al., 2013; Koricheva and Gurevitch, 2014; Gerstner et al., 2017; Gurevitch et al., 2018). Several meta-analyses have been conducted to determine the impact of arbuscular mycorrhizae (AM) on the plant response to salinity stress. In a meta-analysis of 43 studies, Chandrasekaran et al. (2014) reported that AMF enhanced plant biomass and uptake of K and reduced uptake of Na and that the fungal species and the host functional groupings were important moderators of that effect. A recent meta-analysis by Pan et al. (2020) showed that AMF help halophytes increase the accumulation of inorganic ions (K and Ca) and decrease the accumulation of osmolytes (proline and soluble sugar) to augment biomass production under salinity stress. In contrast, in glycophytes, AMF increase salinity tolerance by their combined influence on increasing soluble sugar, nutrient acquisition, superoxide dismutase, and chlorophyll synthesis and decreasing sodium uptake. It was shown that AMF increased the performance of photosystem II in plants under salinity stress by improving the utilization of photons and electron transport and reducing photoinhibition. Under salinity stress, C4 species had better photosynthesis performance than C3 species when inoculated with AMF, and the annual, monocotyledon, and woody species showed better tolerance than plant types (Wang et al., 2019). Augé et al. (2014) carried out a meta-analysis to focus on osmotic and ionic adjustments in AMF plants under salinity. They reported that AMF increased root and shoot K^+^ concentrations, K^+^/Na^+^ ratios, and soluble carbohydrates but had no consistent effect on glycine betaine, Cl^−^ concentrations, leaf Ψ_π_, shoot proline or polyamine concentrations. However, in most studies, the results from the non-stressed condition have not been examined to compare the effects. More importantly, a systematic categorization of salinity levels was not used in these studies, which might result in inaccurate inferences due to the missing impact of the degree of salinity on the effect size. As salinity increases, mycorrhizal colonization and mycorrhizal dependence in plants decrease (Wang et al., 2018). We did not come across any paper that considered the categorization of salinity level and performed meta-analysis of AMF influence at various salinity levels. Additionally, since plant and fungal identity are important moderators (Chandrasekaran et al., 2014), we wanted to examine whether there remained any phylogenetic relevance of fungi and plants in their interactions under varying levels of salinity stress, which has never been tested before.

**Figure 1:**
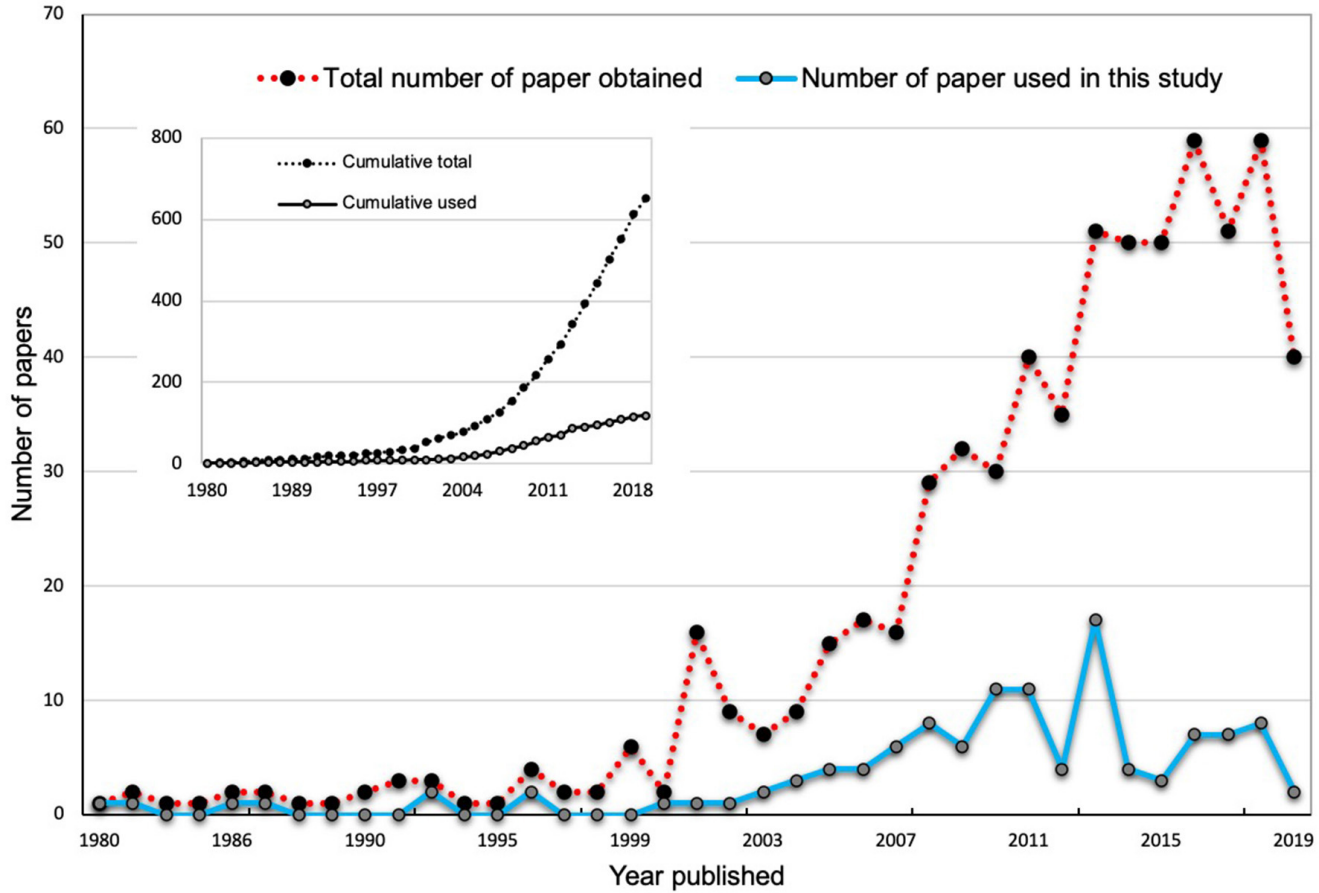
The total number of publications about mycorrhizal effects on plant salinity stress physiology available in the “SCOPUS” and “Web of Science” databases from 1980 to 2019. The inlets in the main plots show the total number of publications found in the search, while the main plots show the number of articles by year.

The publication trend indicates an overall increase in published research on AMF-mediated plant salinity tolerance (Figure 1). Since 2007, there has been a sharp increase in the publication number, indicating an increasing interest in research in this area. However, there remains a paucity of information on the relative importance and magnitude of various factors and mechanisms of AMF-mediated plant salinity tolerance. In the present study, we accumulated data from 97 relevant papers as obtained by our literature search and measured the effects of AMF on 23 plant response parameters encompassing plant growth, photosynthesis, metabolites, and enzymatic activities that are subject to change under salinity stress conditions. We expected that an increased number of articles (97) in our studies as opposed to 43 studies in Chandrasekaran et al. (2014) and 60 studies in Chandrasekaran et al. (2016) would increase the statistical power and robustness in the analyses and accuracy of inferences. The aims of the current study were to answer the following questions:

1. What is the overall impact of AMF colonization on the growth, biomass, nutrient uptake, water relation, and photosynthesis of plants grown under normal conditions as well as those exposed to salinity stress?
2. Do the magnitude and duration of salinity imposition alter the impact of AMF on plants?
3. Is the outcome AMF-plant interaction influenced by various host factors (host species, family, photosynthetic type, lifecycle, lifestyle, nodulation) and symbiont factors (AMF genus), and is the outcome depends on the level of stress?
4. Does AMF effect on plant stress tolerance show any signaling on the phylogeny of AMF or plant at non-stress vs. stressed conditions?
5. What is the relative importance of different antioxidant enzymes (such as CAT, SOD, POD) and osmolytes (proline and sugars) in AMF-mediated plant stress tolerance, and does it vary with the magnitude of stress?

## Materials and Methods

### Literature Search Study Selection

**Figure 2:**
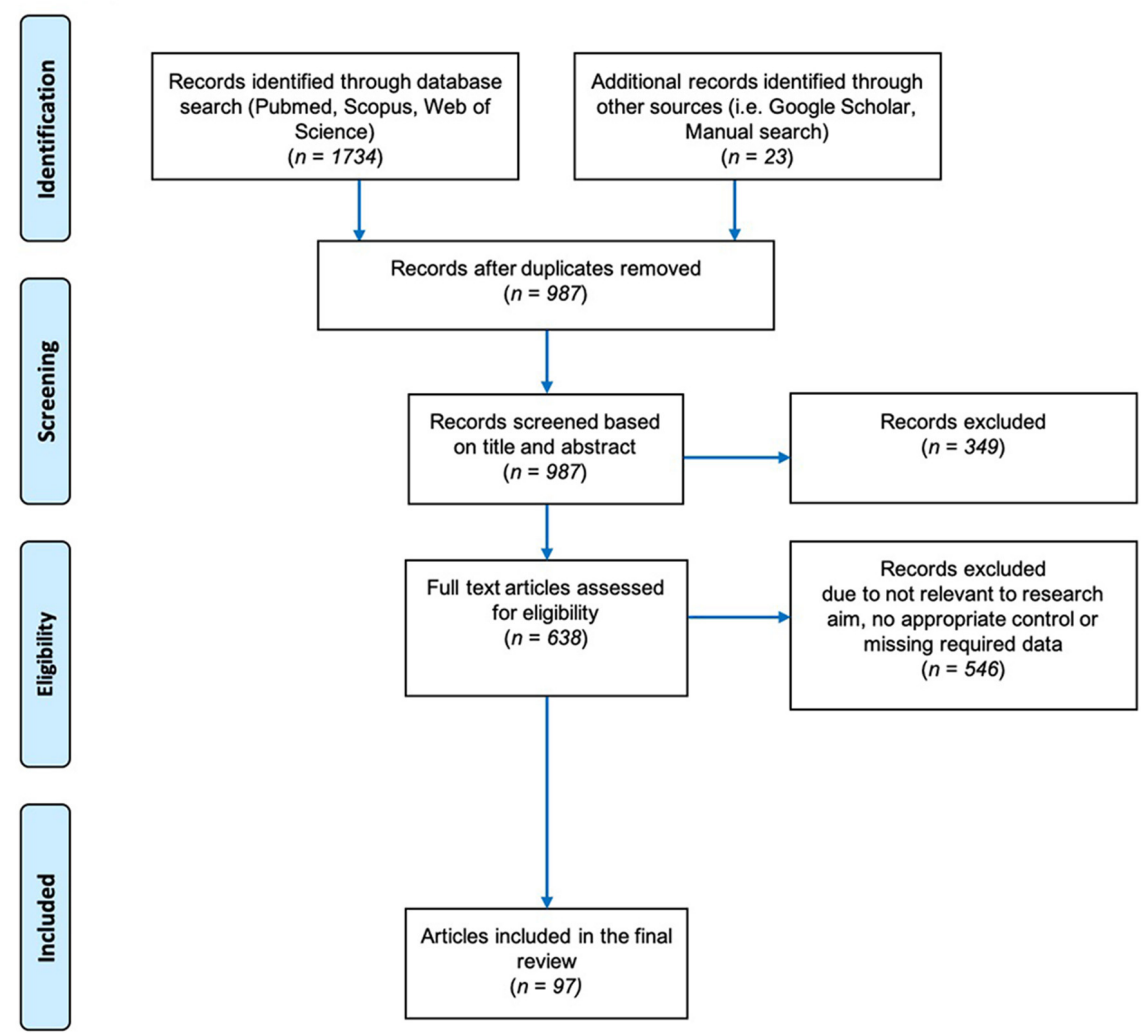
Preferred Reporting Items for Systematic Reviews and Meta-Analysis (PRISMA) Flow Chart describing the search protocol utilized to identify and select published research for this analysis.

The general guidelines of Field and Gillett (2010) were followed in gathering the data for the meta-analysis. We performed a literature search in the Web of Science (Clarivate Analysis) and Scopus databases through May 2019. Our search terms were mycorrhiza^*^ AND salt stress/or under salinity stress, arbuscular mycorrhiza^*^ AND salinity stress/or under salt stress, AMF AND salinity stress/or under salt stress and mycorrhiza^*^/or arbuscular mycorrhiza^*^ plant growth under salt stress. The Boolean truncation (“^*^”) was used to include variations of the word “mycorrhiza” such as mycorrhizae, mycorrhizas, and mycorrhizal. Out of all the search results obtained, 638 were considered likely to contain relevant information based on their title and/or abstract (Figure 2). To make the final selection of the articles for data collection, we used the following set of criteria:

- The experiment had to manipulate at least one AMF strain irrespective of inoculation method or colonization rate,
- The AMF inoculum was used singly, and we avoided mixed inoculation in this analysis,
- Both AMF-inoculated and non-inoculated plants were grown under salinity stress and non-stress conditions,
- Any physiological parameter, e.g., biomass, enzymes, metabolites, etc., was measured, and
- The findings reported sample size, means, standard deviations/errors, and other relevant statistical information such that the outcome could be converted to a standardized measure of effect size.

Of all the studies found, most were rejected based on these criteria, and the list was refined to 97 articles (ST1). We allowed variation among the studies in our meta-analysis in terms of the levels of fertilizer applied, the growth conditions (greenhouse, growth chamber, or field), the duration of time before stress was applied, and the growth media. The papers spanned 39 years (1980–2019) and were in English.

### Data Extraction

From these selected articles, we extracted information on plant biomass, AMF identity, photosynthetic parameters, enzyme parameters, and other relevant data (ST2). The means, sample sizes (replications), and standard deviations were recorded from each study. If standard errors (SEs) were presented, we converted them to standard deviations with the equation SD = SE × √(sample size). The 95% CIs (confidence intervals) reported were converted to SDs where necessary (Vohník et al., 2005). Often, the results were presented in a graph, and we used WebPlotDigitizer V4.2 (https://automeris.io/WebPlotDigitizer/) to digitize the values. Multiple treatments or host/AMF combinations from the same paper were regarded as separate studies and included as independent data units in the analysis. Extracting multiple studies from one experiment might increase the dependence on that study by assuming that the studies are independent (Gurevitch and Hedges, 1999). To examine the potential biases of publication due to non-independence from multiple observations, we calculated the mean effect size of the dataset considering only one random observation from each study and compared this with the effect size calculated with the whole dataset (He and Dijkstra, 2014). We compared effect sizes (full dataset vs. reduced dataset) using Welch's *t*-test to determine whether data reduction could significantly change the effect size. We did not observe any significant discrepancy due to data reduction, indicating that overrepresentation was less likely to occur in this study (ST3). We considered multiple observations as independent since this is thought to increase the statistical power of meta-analysis (Lajeunesse and Forbes, 2003). This approach has been used in various biological meta-analyses (Holmgren et al., 2012; Veresoglou et al., 2012; Mayerhofer et al., 2013; McGrath and Lobell, 2013; Eziz et al., 2017; Dastogeer, 2018).

### Meta-Analysis

Meta-analyses were conducted using the “*meta*” package of Balduzzi et al. (2019) implemented in R version 4.14.0 (R Core Team, 2020). We calculated the standardized mean difference (SMD) using Hedge's *g* statistic to measure the effect size for the difference between means, which is implemented in the “*metacont*” function by default. Hedge's *g* expresses the difference of the means in units of the pooled standard deviation and is preferred in meta-analysis, as it has a lower Type I error rate than other measures, such as the log-response ratio (LRR) (Lajeunesse and Forbes, 2003; Van Kleunen et al., 2010; Xie et al., 2014). The SMD is suggested in meta-analyses that involve studies reporting continuous outcomes, which was the case in our study (Faraone, 2008). An SMD of zero means that the two treatments (AMF-treated or non-treated) have equivalent effects; SMDs >0 indicate the degree to which the AMF-inoculated samples outperformed the non-inoculated samples, and *vice versa*. In general, SMD values of 0.3, 0.5, and 0.8 are interpreted to indicate small, medium, and large effect sizes, respectively (Cohen, 1988). A random-effects model was used to estimate the overall effect. A random-effects model was chosen because of the large number of diverse studies examined, and the studies were not anticipated to estimate a common effect size due to variable locations, conditions, experimental setups and methods used in the individual studies (Borenstein et al., 2011). We assumed that the differences among comparisons and among studies were not only due to sampling error but also due to true random variation, as is common for ecological data (Leimu et al., 2006). The effect size (SMD) was considered significant when the 95% CIs did not include zero. To estimate the random effects variance, the Sidik-Jonkman estimator (Sidik and Jonkman, 2005) was used with Hartung-Knapp adjustment (HKSJ) to make statistical inferences. This approach constructs the confidence interval based on the *t*-distribution and has been shown to improve coverage probability compared to the DerSimonian and Laird (DL) method (Hartung and Knapp, 2001a,b; IntHout et al., 2014). HKSJ produces inflated error rates when the combined studies are of unequal size and show between-study heterogeneity, but it outperforms the widely used DL method (Sidik and Jonkman, 2007; IntHout et al., 2014). To quantify the heterogeneity and to test for statistical heterogeneity, Higgin's I^2^ and Cochran's Q statistics were used, respectively. The I^2^ statistic is defined as the ratio of true heterogeneity to total heterogeneity across the observed effect sizes, while Q represents the weighted deviations from the summary effect size that are due to heterogeneity rather than to sampling error (Higgins and Thompson, 2002; Higgins et al., 2003; Huedo-Medina et al., 2006). I^2^ values range from 0 to 100%, and by convention, values of <25, 25–75, and >75% represent low, moderate, and high heterogeneity, respectively (Higgins et al., 2003). When the homogeneity statistic *Q* was found to be significant (*P* < 0.05 when tested against a chi-square distribution), the data were considered to be heterogeneous and further analyzed by single factor categorical analyses (Mayerhofer et al., 2013).

### Publication Biases and Correction

We tested the publication bias for each dataset with different parameters. We visually inspected asymmetry in funnel plots, used “trim-and-fill” analysis and performed Begg and Mazumdar rank correlation tests based on Kendall's tau, the Egger regression test and p-curve analysis (Begg and Mazumdar, 1994; Egger et al., 1997; Simonsohn et al., 2014) to examine publication biases in the datasets. All these statistics suggested that there were substantial publication biases in some datasets (ST 3). If these tests indicated a bias, then we determined the effect sizes (SMD), CIs, and heterogeneity statistics after applying the trim-and-fill method to correct the biases. Thus, the trim-and-fill altered (decreased) the SMD values on average by 34% (12–48%) compared with the untrimmed SMD values (ST 3). After correcting for publication bias with trim-and-fill, we created subgroups from the studies based on the moderator subgroups and applied trim-and-fill to the subgroups if biases were identified in any of the subgroups by the tests mentioned above (Schmidt and Hunter, 2015). The “trim-and-fill” method is the most widely used method for assessing publication bias in meta-analyses (Duval and Tweedie, 2000a, b; Murad et al., 2018; Shi and Lin, 2019). This approach has so far been very seldom used in plant ecology meta-analyses. However, Nakagawa and Santos (2012) recommended the modification of funnel plots with the “trim-and-fill” method (Duval and Tweedie, 2000b), which allows one not only to test for but also to adjust for publication bias.

### Subgroup Analyses

Subgroup analyses were performed on the data to determine the influence of factors such as plant or AMF identity, plant lifecycle, and salinity duration on the shoot and root dry biomass parameters because sufficient data were available. We ran a mixed-effects model that included the subgroups as the fixed-effects factor using the “*dmetar*” package in R (Harrer et al., 2019). In this model, the overall effect size for each subgroup was calculated using a random effects model where the variance of the summary effect for *k* studies is estimated as

${\text{V}}_{M}=\;\frac{s^{2}}{n}+\;\frac{T^{2}}{k}$ (Borenstein and Higgins, 2013)

Then, we tested between subgroup differences using a fixed effects model where the variance of the summary effect for *k* studies is estimated as

${\text{V}}_{M}=\;\frac{s^{2}}{n}$ (Borenstein and Higgins, 2013).

where n is the cumulative sample size across all studies, s is the standard deviation, *T*^2^ is the estimated variance of effects across studies, and k is the number of studies.

This model is applicable when the subgroup levels under consideration can be assumed to be exhaustive for the characteristic and are not randomly chosen. Most of the subgroups in our study were fixed, such as the plant lifecycle (annual, perennial, or annual/perennial), photosynthesis (C3 or C4) or plant clade (monocot or dicot); therefore, we assumed a mixed effects model to be an appropriate choice. For a factor to be included in the analysis as a subgroup variable, it had to be reported in at least five studies across at least two different articles.

*Mycorrhizal genera*: Nine genera were included in the analyses, namely, *Claroideoglomus, Diversispora, Funneliformis, Gigaspora, Paraglomus, Rhizoglomus, Rhizophagus, Septoglomus*, and *Sieverdingia*. If enough data were not available for some of these genera, they were combined into “other genera” for inclusion in the subgroup analyses.

*Plant family*: There were 18 families included in the analysis. When enough data were not available for some of these families, they were combined as “other” for inclusion in the subgroup analyses. *Plant clade* comprised two levels: *eudicots* and *monocots*. *Plant life cycle*: Plants were categorized by life cycle as *annual, perennial* or *annual/perennial*. Plants that can live annually or perennially were included in the group *annual/perennial*. *Plant lifestyles:* This categorical variable classified plants into *herbaceous* and *woody* plants. *Plant life forms* were grouped into five groups: *forbs, grasses, shrubs, trees*, and *other*. Plants that can have variable life forms, such as shrubs/trees and forbs/shrubs, were included under “other.” We also conducted subgroup analyses based on the nitrogen-fixing ability of plants (legumes vs. non-legumes) as well as the photosynthetic pathway used by the plants (C3 vs. C4).

*Duration of salinity*: We considered the duration of salinity treatment to be short (<2 weeks), moderate (2–4 weeks), or long (>4 weeks). *Salinity level* was defined as three categories: *Low* salinity (<100 mM NaCl or <10 dS/m), *Moderate salinity* (100–200 mM NaCl or 10–20 dS/m), and *High salinity* (>200 mM NaCl or >20 dS/m). The categories of imposed soil salinity were established based on the reported salinity in the published studies.

### Analysis of Phylogenetic Signal

To evaluate whether the effect of AMF symbionts on plant growth was based on the phylogeny of the fungi or plants, we calculated effect sizes for each plant species and fungal species and used each effect size as a trait value to identify phylogenetic signals. For plant phylogenetic signal analysis, we first created a phylogenetic tree by using the R package “V. PhyloMaker,” which is freely available at https://github.com/jinyizju/V. PhyloMaker (Jin and Qian, 2019). For fungal phylogenetic signal analysis, we created alignment using the 18S gene for mycorrhizal species and created maximum likelihood phylogenetic trees using MEGA X version 10.1.5 (Stecher et al., 2020). We based the phylogenies on the 18S region because it is sufficiently variable to distinguish among species, is the most commonly used molecular marker for studying AMF communities, and has a good balance between conserved and hypervariable regions (Öpik et al., 2013; Thiéry et al., 2016). We calculated phylogenetic signals using the R package “phylosingal” (Keck et al., 2016). We computed phylogenetic signal indices such as Abouheif's C mean (Abouheif, 1999), Moran's I (Moran, 1950), Pagel's Lambda (Pagel, 1999), K and K.star (Blomberg et al., 2003) and their corresponding *p*-values using the R packages “adephylo” (Jombart and Dray, 2010), “ape” (Paradis and Schliep, 2019) and “phylobase” (Hackathon et al., 2020). The C mean is designed to detect phylogenetic autocorrelation in a quantitative trait (Abouheif, 1999). Moran's I is a measure of spatial autocorrelation (Moran, 1950) that was adapted for use in phylogenetic analyses by Gittleman and Kot (1990). They refer to it as an autocorrelation coefficient that describes the relationship of cross-taxonomic trait variation to phylogeny.

## Results

We examined the influence of AMF on 23 plant response parameters at varying levels of salinity stress. The summary effect sizes for non-stressed plants in the studies were also considered for comparison. Plant hosts were represented by 51 species in 47 genera and 18 families across the 97 articles and a total of 2,555 experiments for 23 plant parameters. *Zea mays* (9%)*, Solanum lycopersicum* (9%)*, Cajanus cajan* (8%), and *Cicer arietinum* (7%) were the most commonly studied hosts (SF1A). Out of the 15 species of nine fungal genera recorded in all the studies, the most studied fungal species were *Funneliformis mosseae* (36%), *Rhizophagus intraradices* (23%), and *Rhizophagus irregularis* (11%) (SF1B). We used the latest fungal names as described in the Mycobank database (http://www.mycobank.org/).

### Effects of AMF on Plant Growth Parameters Under Salinity Stress

#### Effects of AMF on Plant Shoot Biomass

**Figure 3:**
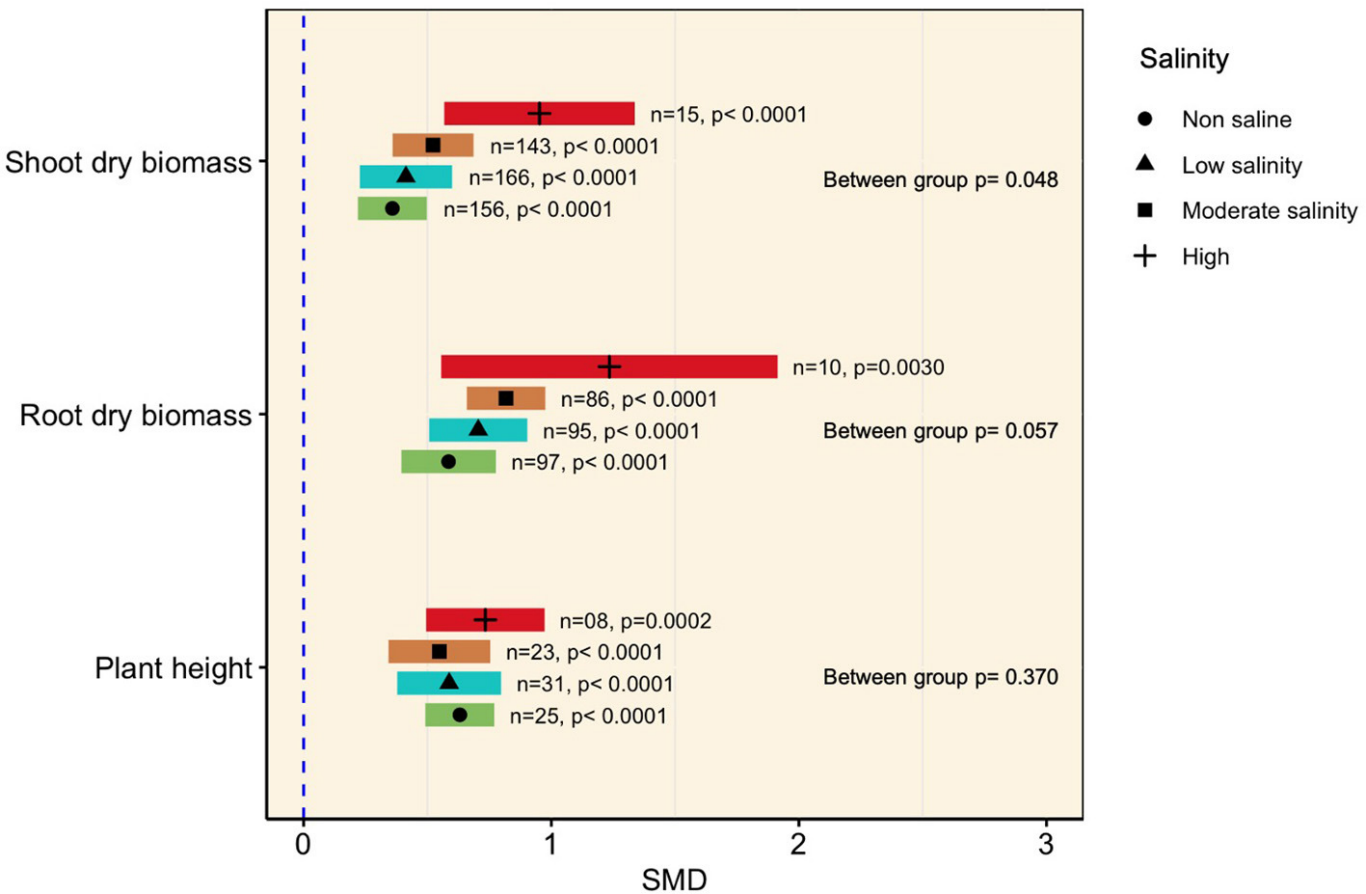
Growth responses of AMF-inoculated plants compared with those of non-inoculated plants under non-stressed conditions and at various levels of salinity. Error bars are effect size (SMD) means ±95% CIs. Where the CIs do not overlap the vertical dashed lines, the effect size for a parameter is significant, i.e., the growth responses of AMF plants were significantly different from those of non-AMF plants. *n*, number of studies included in the meta-analysis; *p*, significance level of SMD.

Generally, mycorrhizal colonization significantly increased plant shoot biomass as the amount of saline increased (*p* < 0.0001, Figure 3). The subgroup analysis revealed that although the effect sizes under moderate (SMD = 0.523) and low salinity (SMD = 0.413) were relatively higher than those under normal conditions (SMD = 0.358), they were not significantly different from each other, as evident from the overlapping confidence interval values (Figure 3). At higher levels of salinity (>200 mM NaCl), however, the influence of mycorrhizae was more prominent (SMD = 0.952) in accumulating shoot biomass than under normal conditions, as apparent from non-overlapping CI values (Figure 3). A similar tendency was evident in the case of plant root biomass, which was substantially increased by AMF association regardless of the level of salinity imposed. It was also observed that the effect size increased with increasing salinity level (the SMDs at non-saline and low, moderate, and high salinity conditions were 0.585, 0.705, 0.817, and 1.25, respectively), but subgroup meta-analysis did not reveal any differences among the effect sizes at various salinity levels (*p* = 0.057, Figure 3). The higher confidence interval values of the effect sizes at higher salinity levels indicated higher variability of mycorrhizal colonization on plant shoot and root biomass accumulation of AMF inoculation had a significant positive impact on plant height under non-stress as well as salt stress conditions. However, no apparent differences in plant height were noticed among the salinity levels (Figure 3, Table 1).

**Table 1:** Heterogeneity statistics for the three biomass summary effect sizes under non-stressed and salinity stress conditions.

**Biomass**	**Salinity level**	**tau^**2**^**	**tau**	**I^**2**^**	**H**	**Q d.f. *p*-value**
Shoot dry biomass	Non-saline	0.5311 [0.0802; 0.4572]	0.7288 [0.2831; 0.6761]	30.3% [14.8%; 43.1%]	1.20 [1.08; 1.33]	222.521550.0003
	Low salinity	1.1841 [0.5051; 1.2423]	1.0882 [0.7107; 1.1146]	54.7% [46.0%; 62.0%]	0.49 [1.36; 1.62]	363.90165 <0.0001
	Moderate salinity	0.6901 [0.1700; 0.6693]	0.8307 [0.4123; 0.8181]	39.5% [25.8%; 50.6%]	1.29 [1.16; 1.42]	234.62142 <0.0001
	High salinity	0.3338 [0.0000; 0.9545]	0.5777 [0.0000; 0.9770]	33.3% [0.0%; 64.1%]	1.22 [1.00; 1.67]	20.99140.1019
Root dry biomass	Non-saline	0.6366 [0.2014; 0.7278]	0.7979 [0.4488; 0.8531]	54.9% [43.2%; 64.1%]	1.49 [1.33; 1.67]	212.6496 <0.0001
	Low salinity	1.6824 [0.6516; 1.8343]	1.2971 [0.8072; 1.3544]	56.3% [46.7%; 64.1%]	1.51 [1.37; 1.67]	290.38127 <0.0001
	Moderate salinity	0.6160 [0.1298; 0.6368]	0.7849 [0.3603; 0.7980]	42.5% [27.8%; 54.2%]	1.32 [1.18; 1.48]	191.35110 <0.0001
	High salinity	0.7452 [0.0000; 4.2383]	0.8632 [0.0000; 2.0587]	44.0% [0.0%; 74.1%]	1.34 [1.00; 1.97]	14.2880.0749
Plant height	Non-saline	11.4567 [3.5176; 21.1810]	3.3848 [1.8755; 4.6023]	63.7% [47.6%; 74.8%]	1.66 [1.38; 1.99]	90.8533 <0.0001
	Low salinity	96.2122 [40.4184; 186.5957]	9.8088 [6.3575; 13.6600]	81.7% [74.8%; 86.7%]	2.34 [1.99; 2.74]	164.0030 <0.0001
	Moderate salinity	4.8334 [0.7138; 8.7916]	2.1985 [0.8449; 2.9651]	49.8% [24.5%; 66.7%]	1.41 [1.15; 1.73]	61.80310.0008
	High salinity	28.7921 [1.9750; 140.6920]	5.3658 [1.4053; 11.8614]	62.9% [23.8%; 82.0%]	1.64 [1.15; 2.36]	21.5980.0057

*Q, total heterogeneity; p, significance of Q heterogeneity; I^2^m, percentage of heterogeneity due to true variation in effect sizes*.

#### Categorical Analysis of the Effects of AMF on Plant Shoot Biomass

**Figure 4:**
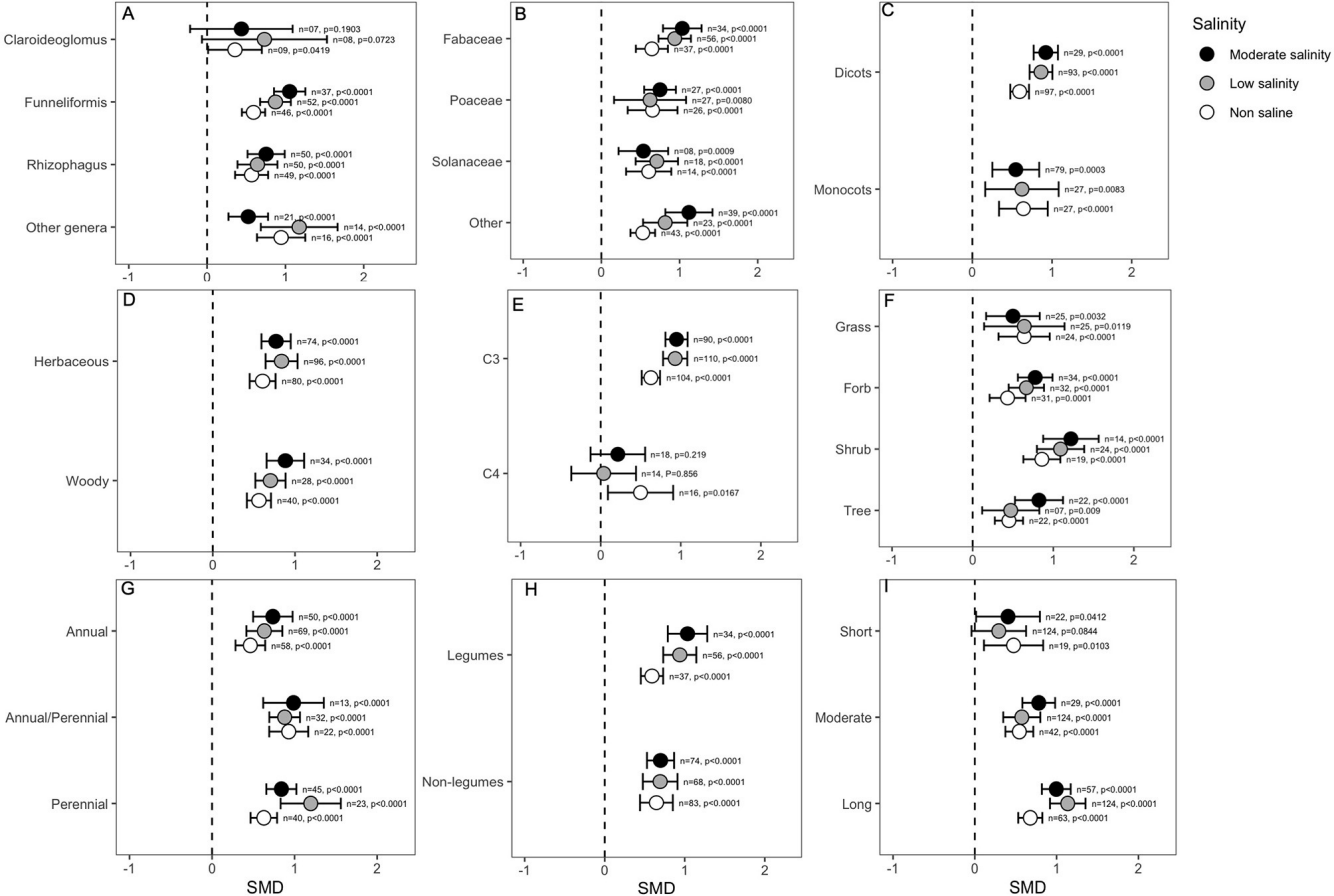
Effects of mycorrhizae on plant shoot biomass under non-saline, low salinity, and moderate salinity conditions for various categorical variables such as **(A)** Fungal genera, **(B)** Plant Family, **(C)** Plant clade, **(D)** Plant lifestyle, **(E)** Plant Photosynthetic pathway, **(F)** Plant Life forms, **(G)** Plant lifecycle, **(H)** Plant nodulation, and **(I)** Salinity duration. Error bars are the effect size means ±95% CIs. Where the CIs do not overlap the vertical dashed lines, the effect size for a parameter is significant, i.e., the growth responses of AMF plants were significantly different from those of non-AMF plants. *n*, number of studies included in the meta-analysis; *p*, significance level of SMD.

Categorical variables considered in the analysis indicated that the effect of mycorrhizal colonization on plant shoot biomass is influenced by several factors, such as host factors, fungus factors and salinity stress. For example, the effect of *Claroideoglomus* on plant shoot biomass was only marginally significant under non-saline conditions (*p* = 0.04), but it did not influence plant shoots when hosts experienced salinity stress. In contrast, other AMF genera, including *Funneliformis* and *Rhizophagus*, showed highly significant effects on biomass in either the absence or presence of NaCl in soil (Figure 4A). Importantly, the effect of *Funneliformis* fungi was much greater at the moderate salinity level than at the normal salinity level (Figure 4A). In addition, relatively narrower CI values for *Funneliformis* implied that mycorrhizae that belong to this genus augmented plant shoot biomass, which is less influenced by other factors, such as host type or salinity level and duration, recorded in different studies. The plant family was not found to impact the AMF response, and the shoot biomass of all plants significantly increased under AMF colonization, although the effect sizes (SMDs) for the *Fabaceae* group were generally higher than those for the other plants (Figure 4B). Plants of the *Fabaceae* family and those included in the “other” group showed a significantly higher influence at moderate salinity than in the absence of salt stress. Both dicot and monocot plants were significantly influenced by mycorrhizal colonization under normal and stressed conditions. Both dicots and monocots increased plant shoot biomass irrespective of salinity treatment. For dicots, the influence of AMF increased significantly as salinity was imposed on plants, which is evident from the non-overlapping CIs for the effect sizes under normal conditions and under salinity stress. Moreover, the higher CI values for monocot plants suggested a wide variability of results in the studies (Figure 4C) compared to the narrower range of CIs for dicots (Figure 4C). We did not observe any difference in shoot biomass effects due to the plant lifestyles; both herbaceous and woody plants accumulated significantly more (*p* < 0.0001) shoot biomass in response to AMF inoculation than non-AM plants, irrespective of the salinity status (Figure 4D). The plant photosynthetic pathway was found to be very important, and C3 plants showed consistently increased shoot biomass as a result of mycorrhizal association. Importantly, under salinity stress, AMF had a significantly higher impact on the shoot biomass of C3 plants compared to their impact in the absence of stress. On the other hand, AMF inoculation in C4 plants only showed a positive response for shoot biomass under non-saline conditions (*p* = 0.016), and in the presence of salinity stress, the effect was neutral or absent (Figure 4E). Moreover, the narrower CI range of the effect sizes for C3 plants than for C4 plants regardless of stress level indicated that the outcome of AMF on shoot biomass was less variable for C3 plants (Figure 4E). The plant life form was not very important in determining the impact of AMF on salinity tolerance. As is evident in the figure (Figure 4F), AMF effects on the forbs, shrubs and tree plants tended to increase as salinity increased, but the situation was reversed for grass plants, although the differences were not significant under normal conditions (Figure 4F). The plant life cycle was not an important factor, but the magnitude of AMF effects on plant shoot biomass was more striking for perennial than for annual plants, even though all plants showed significant positive effects regardless of the salinity treatment (Figure 4G). Plants that form nodules with bacteria, i.e., legumes, outperformed the non-legumes as a result of AMF treatment, as evident in their higher effect size (Figure 4H). Interestingly, the AMF effects on legume plants increased when plants were exposed to stress, as evident from non-overlapping CIs of effect sizes at salinity stress with those under normal conditions (Figure 4H). When plants were exposed to salinity stress only for a short period (<2 weeks), the effect was only marginally significant under non-saline conditions. Under salinity, the AMF effect was not visible over a short period, but as the duration of salinity stress increased, the effect continued to increase. When plants were subjected to a long period (>4 weeks) of stress, the impact on AMF on plant shoot biomass was highly significant compared to their effects under normal conditions (Figure 4I).

#### Categorical Analysis of the Effects of AMF on Plant Root Biomass

**Figure 5:**
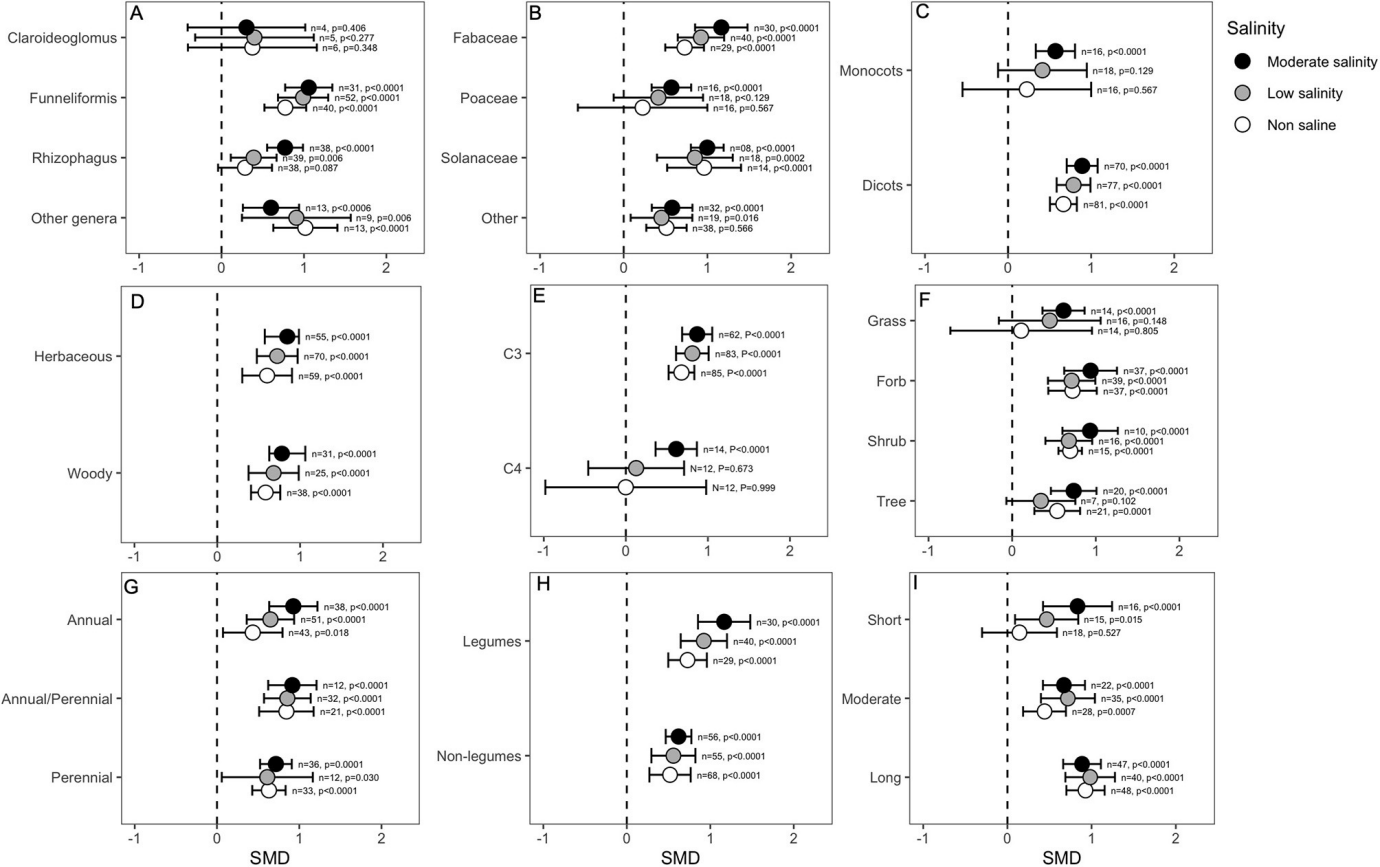
Effect of mycorrhizae on plant root dry biomass under non-saline, low salinity, and moderate salinity conditions for various categorical variables such as **(A)** Fungal genera, **(B)** Plant Family, **(C)** Plant clade, **(D)** Plant lifestyle, **(E)** Plant Photosynthetic pathway, **(F)** Plant Life forms, **(G)** Plant lifecycle, **(H)** Plant nodulation, and **(I)** Salinity duration. Error bars are effect size means ±95% CIs. Where the CIs do not overlap the vertical dashed lines, the effect size for a parameter is significant, i.e., the growth responses of AMF plants were significantly different from those of non-AMF plants. *n*, number of studies included in the meta-analysis; *p*, significance level of SMD.

Most categorical variables considered for analysis indicated that mycorrhizal colonization differentially influenced plant root biomass. For example, the fungal genus *Claroideoglomus* seemed to have no substantial effect on root biomass under any conditions. In contrast, *Funneliformis* and the genera classified into the “other” category also had positive effects on root mass under normal as well as salinity stress conditions. Interestingly, *Rhizophagus* increased root growth only in the presence of salt stress (Figure 5A). The root biomass of poaceous plants was influenced positively only under moderate salinity stress conditions. Nevertheless, other plants, such as those belonging to Solanaceae and Fabaceae, had consistently higher root mass in the AMF-inoculated plants under both normal and stressed conditions (Figure 5B). The plant clade seemed to be a crucial determining factor for the AMF response. Dicots showed significantly higher (*p* < 0.0001) root growth in the AMF-inoculated plants irrespective of the salinity conditions, whereas monocot roots responded positively to AMF only at higher salinity levels (Figure 5C). Overall, the magnitude of effect sizes was not much different between woody vs. herbaceous plants, and the roots of both groups were influenced positively by AMF treatment (Figure 5D). The C3 plants had, in general, very high SMD values (~1.00), suggesting a positive correlation between AMF inoculation and root growth. In contrast, the root biomass of C4 plants was not influenced by AMF as the salinity increased to a moderate level (*p* < 0.0001), and the plants received benefits from the fungi and increased their root mass (Figure 5E). Grasses were less influenced by AMF treatment than forbs and shrubs, which showed significantly higher root mass in the colonized plants (Figure 5F). The life cycle of the plant did not strongly influence the response to AMF, and both annual and perennial plants showed higher root mass in the presence of AMF regardless of the salinity stress level. Plants that are perennial but that are generally cultivated as annuals had higher SMD values than other plants (Figure 5G). Nodulating plants had overall higher effect sizes (~1.00) for root biomass than non-nodulating plants (~0.600), but in both groups, root mass significantly increased in AMF-treated plants compared to that in non-treated plants under all salinity conditions (Figure 5H). Notably, when the roots were harvested after a relatively longer period of AMF treatment (>4 weeks), the effect of AMF on increasing root biomass was highly significant regardless of the salinity condition. When the duration was <4 weeks but >2 weeks, AMF plants still had higher root mass than non-AMF plants, and salinity-stressed plants experienced a greater increase in root mass than non-stressed plants. If the treatment was very short (<2 weeks), the AMF effect on plant roots was neutral under normal conditions, but it became significant as the salinity increased (Figure 5I).

#### Phylogenetic Signal of AMF-Mediated Biomass Modulation Under Stress

**Figure 6:**
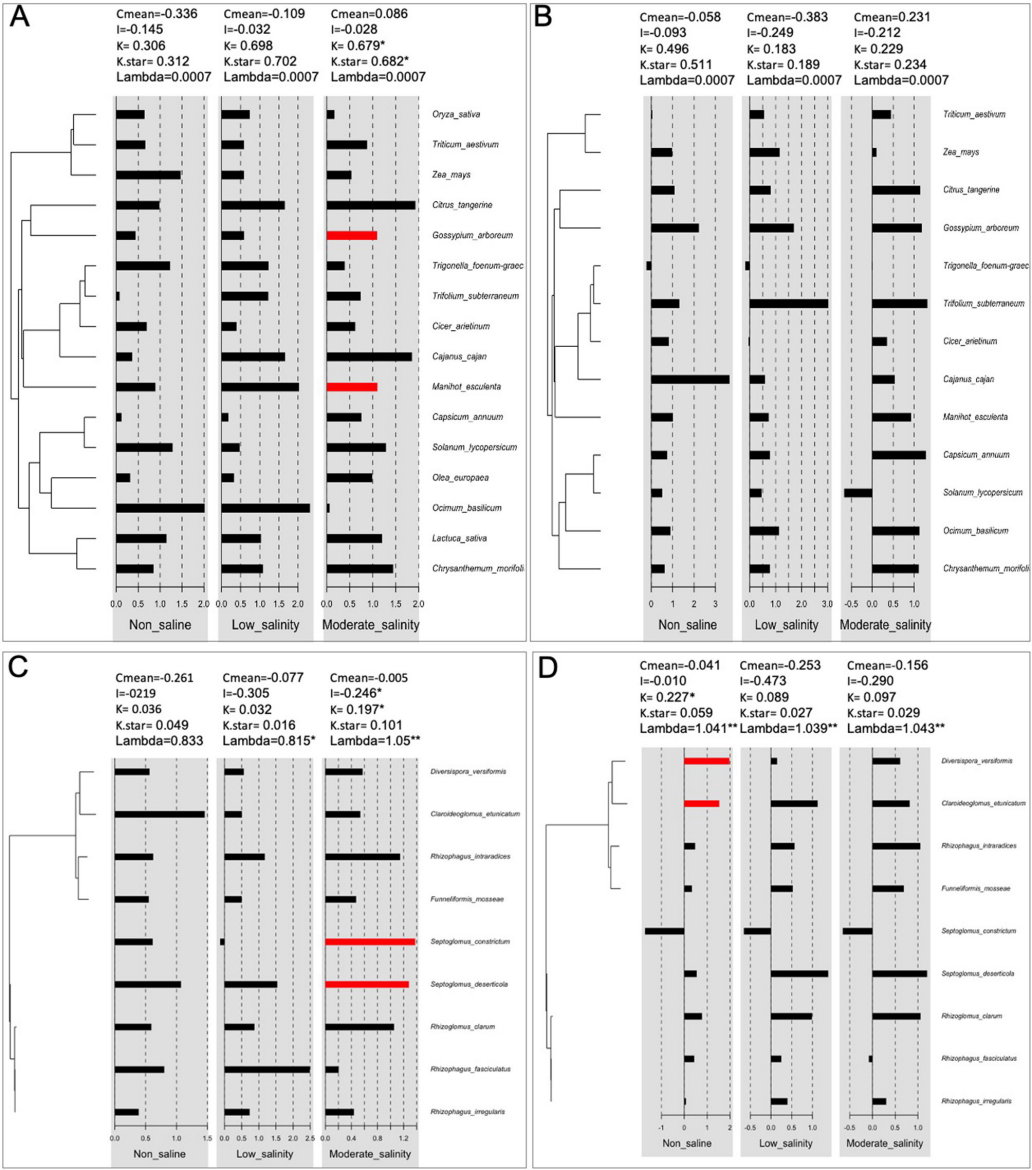
Phylogenetic signals of plant families **(A,B)** and fungi **(C,D)** for the effects of AMF on plant shoot **(A,C)** and root **(B,D)** biomass under non-saline and salinity stress conditions. C mean, I, K, K-star, and lambda are as described in the section Materials and Methods. The red bar indicates that the signals are positive. *, **indicates that the signals are significant at *p* < 0.05 and *p* <0.01, respectively.

We observed a significant plant phylogenetic signal, i.e., closely related plant species had more similar shoot biomass responses to AMF treatment than distantly associated species under moderate salinity stress but not under non-saline or low salinity conditions (Figure 6A). However, the response of root biomass to mycorrhizal association had no relationship with conserved plant phylogeny under any conditions (Figure 6B). Again, mycorrhizae that are more closely related phylogenetically had similar influences on plant shoot biomass under moderate salinity, but for the root mass trait, the phylogenetic signal could be identified only under normal conditions (Figures 6C,D).

### Effects of AMF on Plant Photosynthetic Attributes and Water Status

**Figure 7:**
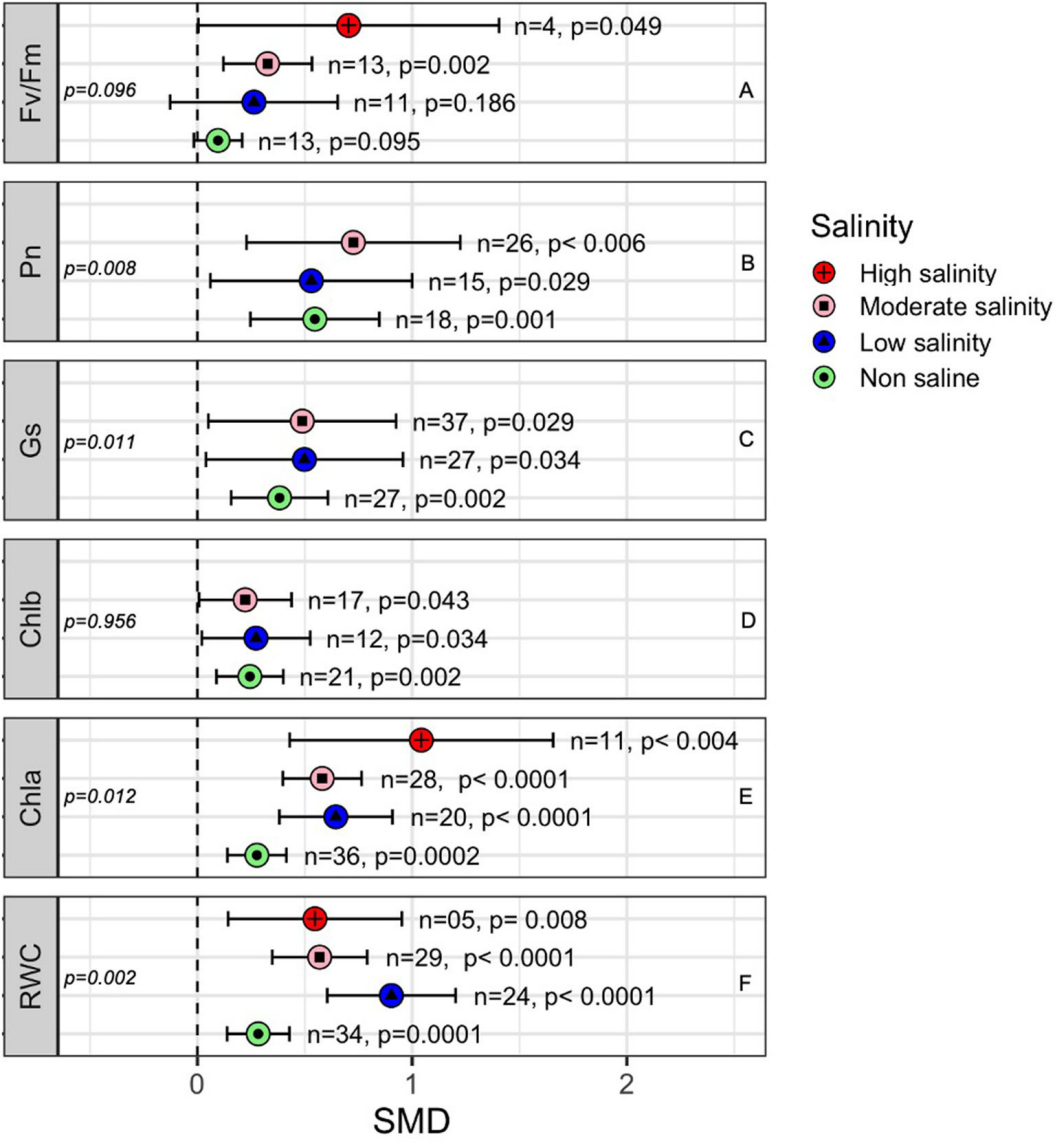
Effects of mycorrhizae on plant photosynthetic parameters under different levels of salinity stress. Error bars are the effect size means ±95% CIs. Where the CIs do not overlap the vertical dashed lines, the effect size for a parameter is significant. *n*, number of studies included in the meta-analysis. Chla, Chlorophyll a; Chlb, Chlorophyll b; Fv/Fm, maximal photochemical efficiency; Gs, Stomatal conductance; Pn, rate of photosynthesis; and RWC, Relative water content; *n*, number of studies included in the meta-analysis; *p*, significance level of SMD.

Most of the photosynthetic parameters, such as the rate of photosynthesis (Pn), stomatal conductance (Gs) and chlorophyll a (Chla), and chlorophyll b (Chlb) content, were significantly influenced by AMF colonization under both stressed and non-stressed conditions (Figure 7). The subgroup analyses showed that the positive effects of AMF on certain plant photosynthetic parameters, especially Chla, Gs, and Pn, were higher when plants were exposed to salinity stress than those in non-stressed plants (Figure 7). For example, the quantum efficiency of PS II (Fv/Fm) was significantly influenced by AMF inoculation under moderate (*p* = 0.002) and high salinity (*p* = 0.049) conditions but not under low salinity or non-saline stress conditions (Figure 7A). The photosynthesis rate of AMF-treated plants was consistently higher than that of non-AMF plants regardless of salinity stress (Figure 7B). Stomatal conductance (Gs) was higher in AMF plants under salinity stress (SMD = 0.488), but it the difference at normal conditions (SMD = 0.382) were not very high in AMF plants than in non-inoculated plants (Figure 7C). Chlorophyll a increased more in AMF plants under salinity (SMD = 0.581 at moderate salinity, 0.644 at low salinity) than under normal (SMD = 0.277) conditions (between-group difference, *p* = 0.012) (Figure 7D). Chlorophyll b, on the other hand, increased with AMF but did not vary significantly due to salinity stress (*p* = 0.956). The leaf relative water content (RWC) in mycorrhiza-inoculated plants was consistently higher than that in non-inoculated plants regardless of the salinity stress level. Under low salinity conditions, the influence of AMF on RWC was significantly higher than under non-saline conditions (Figure 7F). All the parameters considered, however, tended to be more variable under salinity stress than under non-stressed conditions, as is evident from their larger confidence interval values (Figure 7).

### AMF Effects on Plant Nutrient Homeostasis

**Figure 8:**
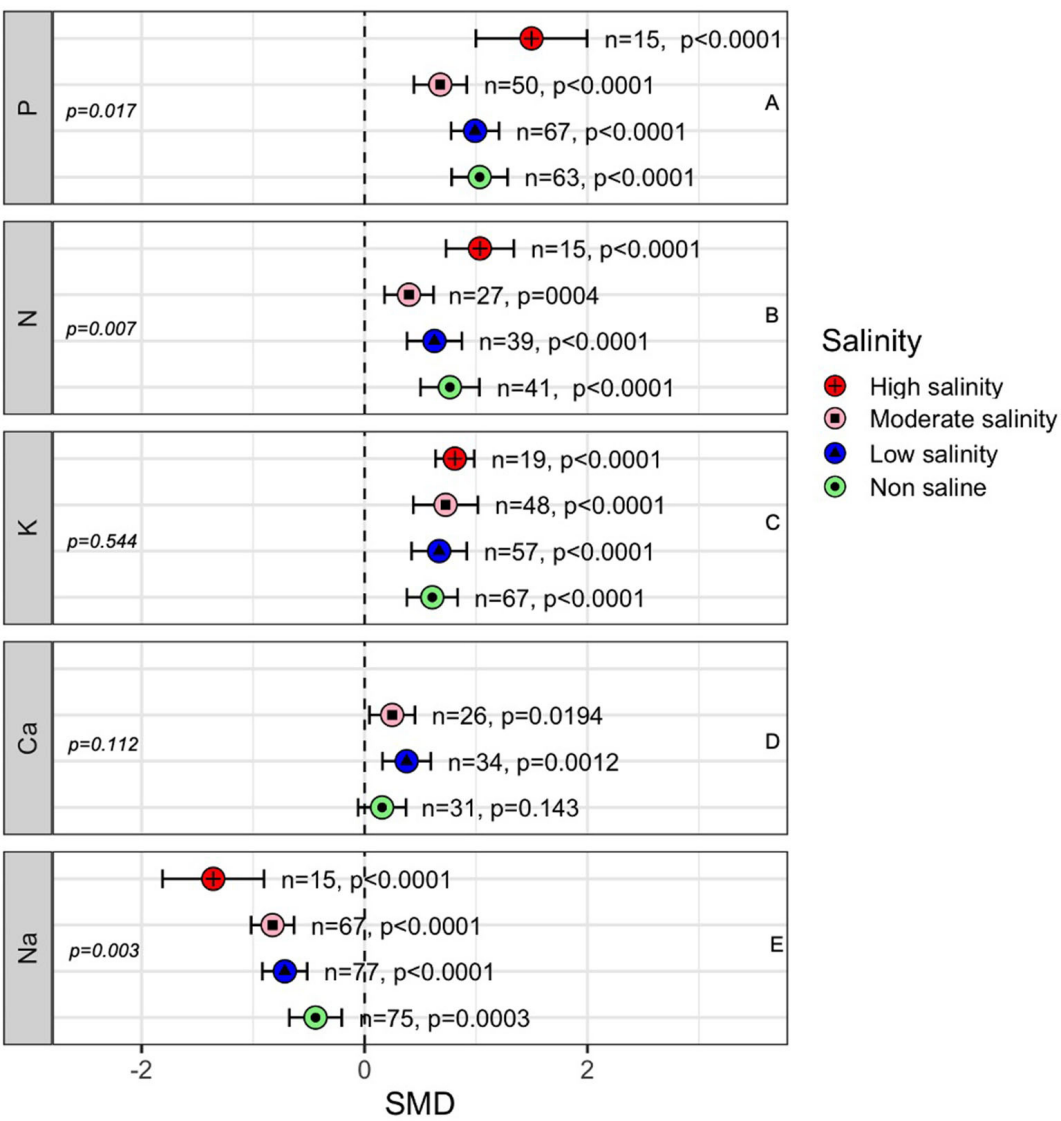
Effects of mycorrhizae on plant nutrient uptake under non-stress, low salinity stress, and moderate salinity stress. Error bars are effect size means ±95% CIs. Where the CIs do not overlap the vertical dashed lines, the effect size for a parameter is significant. *n*, number of studies included in the meta-analysis. Ca, Calcium; K, potassium; Na, sodium; N, Nitrogen and P, phosphorus. *n*, number of studies included in the meta-analysis; *p*, significance level of SMD.

The concentrations of nutrients such as N, P, K, Ca, and Na in the above-ground parts of plants were influenced significantly by AMF colonization under both stressed and non-stressed conditions (Figure 8), indicating the role of AMF in plant nutrient uptake. Plant P uptake showed an increase by AMF regardless of salinity although there was a decreasing trend as the salinity stress increases up to moderate level (Figure 8A). Interestingly, as the salinity stress increased from moderate to high level significantly higher-level P concentrations was measured in AMF plants compared to non-AMF plants (SMD = 0.679 at moderate salinity, 1.50 at high salinity). Like P, we observed a similar trend in N concentration in plant shoots (Figure 8B). Plants inoculated with AMF had consistently higher K levels in the shoot (*p* < 0.0001), and salinity had a slight impact on AMF-induced K uptake (*p* = 0.544) (Figure 8C). The concentration of Ca in the shoot was affected by AMF and salinity. Under normal conditions, AMF inoculation seemed to have an no effect (*p* = 0.143), but as the plants were exposed to a low (<100 mM NaCl) level of salinity, the Ca content in AMF-inoculated plants increased (*p* = 0.001). However, increasing the salinity beyond this level reduced this advantage although still significant (*p* = 0.019) than that in non-inoculated plants (Figure 8D). Plant Na uptake was decreased by mycorrhizal fungal inoculation. The magnitude of the decrease was higher at higher salinity levels (*p* = 0.003), e.g., Na uptake was significantly lower in AMF plants than in non-AMF plants at a high level of salinity compared to normal conditions (Figure 8).

### AMF Effects on Plant Antioxidant and Enzyme Activities

**Figure 9:**
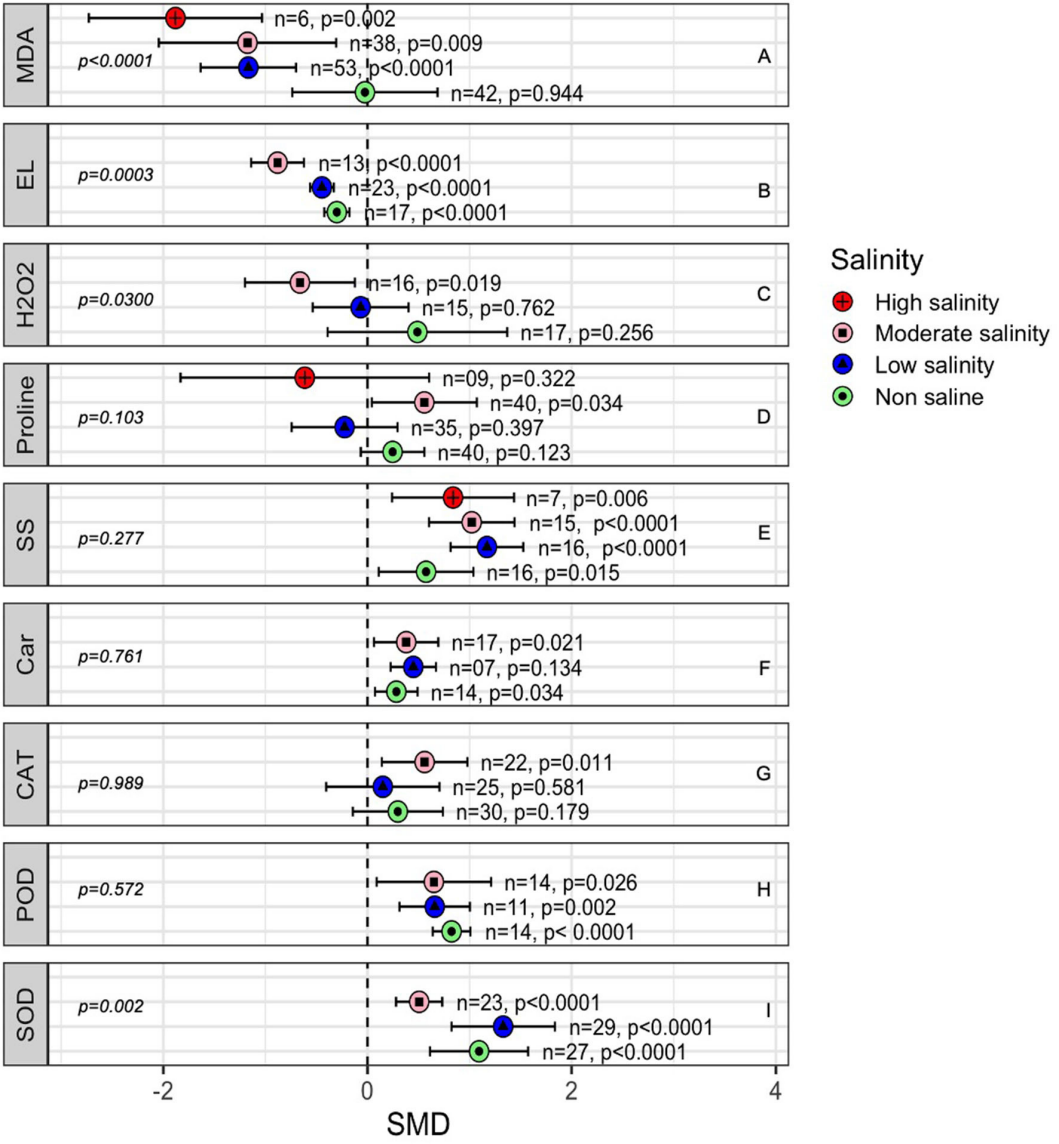
Effects of mycorrhizae on plant enzymatic activity under non-stressed, low salinity, and moderate salinity stress. Error bars are means ±95% CIs. Where the CIs do not overlap the vertical dashed lines, the effect size for a parameter is significant. *n*, number of studies included in the meta-analysis. CAT, catalase; Car, Carotenoids; EL, electrical leakage; H2O2, hydrogen peroxide; MDA, malondialdehyde; POD, peroxidase and SOD, superoxide dismutase and SS, soluble sugars. *n*, number of studies included in the meta-analysis, *p*, significance level of SMD.

The generation of malondialdehyde (MDA) under stress is related to the production of reactive oxygen species, including hydrogen peroxide (H_2_O_2_), in plant tissue. Under non-stressed conditions, there was no difference between AMF- and non-inoculated plants in terms of H_2_O_2_ and MDA (Figures 9A,C) concentrations. However, AMF-inoculated plants had significantly lower MDA levels than non-inoculated plants as the salinity stress increased. The effect of AMF on H_2_O_2_ production in plants was only substantial under moderate salinity (*p* = 0.019, Figure 9). Decreased electric leakage (EL) was consistently measured in AMF-inoculated plants compared with that in non-inoculated plants, but the AMF effects became more apparent (p = 0.0003) as the salinity increased from no salinity to a moderate level of salinity. Proline accumulation was not affected by AMF, but moderate salinity caused a higher level of proline accumulation in AMF-treated plants that was marginally higher (*p* = 0.034) than that in non-AMF-treated plants (Figure 9D). Both carotenoid and soluble sugar contents increased in AMF-treated plants compared with those in non-inoculated plants. No influence of salinity on AMF activity was noted in terms of carotenoid accumulation, but as salinity level increased, the effect size of AMF on the soluble sugar content increased (Figures 9E,F). The activity of catalase (CAT) did not change in plants due to AMF colonization under non-stressed or low salinity conditions, but moderate salinity caused the CAT activity to significantly increase in AMF plants (SMD = 0.559, *p* = 0.026). On the other hand, the activity of POD (peroxidase) and SOD (superoxide dismutase) increased substantially in AM-inoculated plants compared to that in non-inoculated plants. Salinity imposition did not seem to impact AMF influence on POD but on SOD. At moderate salinity significantly lower accumulation of SOD was observed in AMF plants when compared to the level at normal condition than in non-inoculated plants in the absence of salt stress (Figures 9G,H).

## Discussion

### Effects of AMF on Plant Biomass

This study indicated that mycorrhizal inoculation has a significant impact on some plant physiological and biochemical variables related to plant growth, photosynthesis, and defense against oxidative damage under salinity stress.

Salinity induces both osmotic and ion stress in plants. Within a short exposure time, salinity-driven osmotic stress causes water scarcity in the root zone and directly impairs the water status of plants. However, the plant recovers over several hours and reaches a slow, steady rate of growth. The second phase develops with time and is driven by the toxicity of excess Na^+^ and Cl^−^ ions that accumulate in the cytoplasm. Moreover, under salinity stress, plants need additional energy to reduce the toxic effects of Na^+^ ions and also face nutrient deficiencies. All these processes negatively affect plant growth (Munns and Tester, 2008; Ilangumaran and Smith, 2017; Isayenkov and Maathuis, 2019). Mycorrhizal colonization has been shown to increase plant growth and photosynthetic efficiency under stress conditions (Chandrasekaran et al., 2014; Shamshiri and Fattahi, 2016; Elhindi et al., 2017). In the present meta-analysis, we confirmed that AMF inoculation increases the height, shoot biomass, and root biomass of host plants and that, interestingly, the effect of AMF on plant growth is more prominent under salinity stress than under normal conditions. AMF association in plants influences plant shoot biomass and root biomass more than plant height. Again, AMF influences on plant growth were context-dependent, with several factors playing important roles. For example, *Funneliformis* increased both the shoot and root biomass of plants under stress, whereas *Claroideoglomus* increased only the shoot biomass, and *Rhizophagus* influenced only the root biomass. In most cases, the effects of fungi on plant growth parameters became more noticeable as the salinity level increased. This result indicates that the plant growth response to AMF colonization is context-dependent. The beneficial effects of fungal symbiosis increase under extreme environments (Redman et al., 2002; Bunn et al., 2009; Dastogeer, 2018). A meta-analysis with AM fungi and leaf endophytes and plant growth parameters reported similar results; the effects of the fungi increased as moisture stress increased (Worchel et al., 2013). Studying the effects of categorical variables on plant growth will help us to select some potential efficient plant-AMF associations in which AMF inoculation will more strongly modulate plant growth in the presence of salinity. Interestingly, under salinity stress conditions, AMF inoculation increased plant growth traits more efficiently in dicot plants than in monocots. This result was supported by a previous finding that AMF colonization was higher in dicots than in monocots (Weishampel and Bedford, 2006). Similarly, the growth of C3 plants was influenced more than that of C4 plants by AMF inoculation under salinity stress, which corroborates the findings of another meta-analysis (Chandrasekaran et al., 2016). However, out analyses did show not any positive influence of AMF on shoot biomass of C4 plants which somewhat contradictory to the findings of Chandrasekaran et al. (2016). Therefore, based on the present meta-analysis, we can recommend the application of AMF to C3-dicot plants to effectively alleviate salinity-induced growth inhibition. Furthermore, in our meta-analysis, the effect of AMF treatment in plants was more pronounced at longer duration of salinity stress (>2 weeks).

### Water Status and Photosynthesis

All photosynthetic parameters included in the meta-analysis were found to be present at a significantly higher level in AMF-treated plants than in non-AMF plants, and this level increased with salinity stress (Figure 7). AMF can help plants mitigate or reduce the detrimental effects of salt stress on photosynthesis in various ways. As our meta-analysis showed, AMF improved the water status in plants and thus allowed them to maintain a larger leaf area and higher stomatal conductance, which improved the assimilation rate of CO^2^ (Wu et al., 2015; Chen et al., 2017).

The higher water status in AMF-treated plants has been explained by several reports. For example, mycorrhizae can influence root morphology, and with far-reaching extramatrical mycelium, they can acquire macroelements beyond the depletion zone (Schnepf et al., 2011). Moreover, treatment with AMF increases the water use efficiency of plants by augmenting the concentration of compatible solutes to modulate the osmotic potential (Graham and Syvertsen, 1984). Several studies have suggested enhanced RuBisCO enzyme activity in AMF-treated plants, which reduces the intercellular CO^2^ concentration (Ci) to provide better protection to the photosynthetic apparatus (Sheng et al., 2008; Chen et al., 2017). Mycorrhizae help plants reduce the degradation of D1 and D2 proteins under salt stress and thus maintain the function of photosystem II, which is important in adapting to stress conditions (Porcel et al., 2016; Chen et al., 2017; Hu et al., 2017). The presence of more polyamines and glycine betaine has also been reported in AMF plants, which is linked to safeguarding CO^2^-fixing enzymes (Pang et al., 2007; Talaat and Shawky, 2014). We did not include Mg^2+^ data in our study, but several studies have suggested that a higher concentration of this cation is present in AMF-inoculated plants, which may be associated with higher chlorophyll in AMF plants (Evelin et al., 2012; Hashem et al., 2015). In addition, the enhanced photosynthesis in AMF-treated plants could be related to decreased non-photochemical quenching (NPQ) activity and increased Fv/Fm (Baker, 2008; Hu et al., 2017).

### Nutrients

Na^+^ and K^+^ ions, which have similar physicochemical properties, compete at transport sites for entry into the symplast. Higher Na^+^ levels in the rhizosphere reduce K^+^ uptake in plants under saline conditions (Maathuis and Amtmann, 1999). Higher Na^+^ levels affect the integrity and selectivity of the root membrane (Grattan and Grieve, 1999). Plants must consistently maintain a low Na^+^ to K^+^ ratio to tolerate salinity stress (Evelin et al., 2012). AMF-treated plants showed higher K^+^ and lower Na^+^ than non-AMF-treated plants under salt stress conditions (Figure 8). AMF-inoculated plants can affect Na^+^ translocation to the upper plant parts and maintain the internal Na^+^ concentration. Mycorrhizae help plants remove Na^+^ from xylem and prevent its accumulation in photosynthetic tissues (Evelin et al., 2012; Maathuis, 2014). It was reported that salinity induces the accumulation of glomalin, a heat shock protein 60 (HSP60) homolog, in the AMF (Hammer and Rillig, 2011), which decreases the damage caused by Na^+^ in the cytosol (Maathuis and Amtmann, 1999). Several studies have detailed the molecular basis of the high K^+^: Na^+^ ratio in AMF-inoculated plants (Asins et al., 2013; Porcel et al., 2016; Chen et al., 2017). For example, in *Oryza sativa*, mycorrhizae compartmentalize Na^+^ into the vacuole by upregulating OsNHX3 (sodium/hydrogen exchanger) and facilitate the removal of cytosolic Na^+^ to the apoplast through the increased expression of OsSOS1 (salt overly sensitive) and OsHKT2;1 (high-affinity potassium transporter) (Porcel et al., 2016). The higher nutrient uptake in AMF-treated plants is attributed to several factors, including the extramatrical hyphae of mycorrhizae (Marschner and Dell, 1994). AMF colonization influences organic acids and polyamines in plants, which play positive roles in decreasing soil EC, maintaining plant ion homeostasis and enhancing nutrient and water uptake under stress (Pang et al., 2007; Sheng et al., 2011; Evelin et al., 2013; Talaat and Shawky, 2013).

The higher P uptake in AMF-inoculated plants helps maintain membrane integrity by reducing ionic leakage, restricting toxic ions within vacuoles, and enforcing selective ion uptake (Rinaldelli and Mancuso, 1996; Evelin et al., 2012), which consequently reduce the adverse effects of salinity. The increased P uptake in colonized plants has several causes: (a) fungal hyphae secrete acid and alkaline phosphatases that release P and make it available to plants, (b) when P is available, high-affinity phosphate transporter genes (*GvPT, GiPT*, and *GmosPT*) are expressed, which can release P even at very low concentrations, and (c) mycorrhizal roots can obtain higher amounts of absorbed P than non-mycorrhizal roots, resulting in a consistent supply of P into the roots (Bolan et al., 1991; Marschner and Dell, 1994; Selvaraj and Chellappan, 2006; Abdel-Fattah and Asrar, 2012). Moreover, AMF colonization in roots helps improve nutrient uptake, specifically N uptake, with extensive underground extraradical mycelia ranging from the roots into the surrounding rhizosphere (Battini et al., 2017).

### Oxidation and Antioxidants

Salinity stress induces oxidative stress by creating anomalies in the production and destruction of reactive oxygen species (ROS) (Gill and Tuteja, 2010). Salinity stress-induced lipid peroxidation results in uncontrolled membrane permeability and ion loss from the cells (Estrada et al., 2013; Fileccia et al., 2017). Plant malondialdehyde (MDA) levels are measured as a biomarker for lipid peroxidation to evaluate oxidative stress in plants. As salinity increases, MDA levels increase in plants, indicating the level of stress that the plants are experiencing (Asada and Takahashi, 1987; Gill and Tuteja, 2010; Sharma et al., 2012; Ozgur et al., 2013; Bose et al., 2014; Kumar et al., 2017). Our results suggest that AMF-colonized plants have significantly lower MDA levels, indicating less oxidative stress in these plants than in non-AMF plants. Plants use both enzymatic (SOD, POX, CAT, APX, and GR enzymes) and non-enzymatic antioxidative systems [ascorbate (AsA), glutathione (GSH), carotenoids, and α-tocopherol] to detoxify ROS (Evelin et al., 2009; Gill and Tuteja, 2010; Porcel et al., 2012). Our meta-analysis showed that AMF increased the enzymatic activity of plants in the presence of salts. Importantly, POD and SOD activity increased in mycorrhizal plants at low salinity levels. Higher SOD activity is correlated with higher plant tolerance to salinity (Benavídes et al., 2000). SOD helps in the detoxification of excess O^2−^ to H_2_O_2_. H_2_O_2_ is then converted to H_2_O by other enzymes, such as CAT, APX, and POX. Interestingly, some studies showed that mycorrhizal plants have higher CAT activity, but we found that the net effect was neutral when the salinity was low (Figure 6). This suggests that the role of CAT is not as important as that of POD and SOD in AMF-mediated plant salinity tolerance at a lower level of stress, but as the salinity increases, CAT becomes activated, and a combination of these enzymes work toward antioxidation. Again, as noted elsewhere, the activities of these enzymes vary due to factors such as plant species, plant tissues, AMF species, level of salinity, and duration of stress (Evelin et al., 2019). While we did not estimate the magnitude of these factors, the relatively higher CI values of the effect sizes indicate the roles of these factors. One of the indicators of plant damage caused by salinity stress is the relative amount of electrolyte leakage. A higher level of relative electrolyte leakage implies more salinity injury to plant membrane systems (Verslues et al., 2006; Sánchez-Rodríguez et al., 2010; Alqarawi et al., 2014). We showed that electrical conductivity was significantly lower in AMF plants, which suggests lower damage to the cell membrane; electrical conductivity is also correlated with lower MDA levels (Figure 9) but is not correlated with the effect size for H_2_O_2_ production. The lower EL and MDA, as well as the higher POD and SOD, reflect better antioxidant responses that protect plants from oxidative injury in AMF-treated plants than in non-AMF-treated plants. POD is a group of enzymes that can detoxify H_2_O_2_, organic hydroperoxide, and lipid peroxides and convert them to alcohol. They have a haem cofactor at their active sites. Haem is also related to iron homeostasis, which is involved in plant-microbe interactions (Briat et al., 2007). In addition, the redox-active cysteine residues in POD indicate the redox potential of cells or organelles. The plastid modulates the redox potential in leaves (Mühlenbock et al., 2008; Brautigam et al., 2009). Whether fungi interfere with the iron homeostasis and redox potential of the plant cell and increase plant stress tolerance *via* this mechanism has yet to be discovered.

Proline levels increased significantly under moderate salinity conditions in AMF-inoculated plants. Proline can play important roles as an osmoregulatory compound (Yoshiba et al., 1997) as well as an ROS scavenger (Dickman and Chen, 2005). The accumulation of proline is correlated with both osmotic stress tolerance and responses to stress conditions involving dehydration (Aspinall and Paleg, 1981; Gzik, 1996; Verbruggen and Hermans, 2008). However, it is still unresolved whether its presence is an adaptive response that provides greater stress tolerance or if its increase is a symptom of stress injury (Ashraf and Foolad, 2007). Relatively high levels of proline in the presence of AMF could, therefore, indicate less damage to a moderately stressed plant in the presence of AMF.

### Some Recommendations

This systematic review highlights the importance of AMF in mitigating plant salinity stress and the importance of AMF-mediated plant salinity tolerance. Our current analysis, as well as the recent review paper by Evelin et al. (2019), suggests several aspects that need more attention in order to develop a complete and robust understanding of AM-conferred plant salt stress tolerance. We discuss these aspects in the following bullet points:

- The presence of publication biases was apparent in our datasets. Publication bias is a serious issue in meta-analyses that can have profound effects on the validity and generalization of the conclusions (Lin and Chu, 2018). Although publication bias has been discussed in medical and social sciences for several years, in ecology and plant sciences, this discussion has just started in the last few years (Alatalo et al., 1997; Gontard-Danek and Møller, 1999; Palmer, 2000; Møller and Jennions, 2001; Dieleman and Janssens, 2011; Koricheva and Gurevitch, 2014). It has been reported that the majority (61%) of meta-analyses in plant ecology did not show or mention publication bias or mention the term “publication bias” (Koricheva and Gurevitch, 2014). Therefore, it is uncertain how robust the conclusions of these meta-analyses are. Therefore, we, along with many other authors, urge researchers to report publication biases in meta-analysis reports. Additionally, factors that contribute to publication biases such as submission bias, editor bias, reviewer bias, etc., as discussed elsewhere (Møller and Jennions, 2001) should be avoided in the publication of scientific reports.
- Most research has focused on the effects of AMF on plant physiology by inoculating individual inocula or mixtures of only a few strains, and less attention has been paid to discerning the effects of complex inoculum mixtures. In nature, AMF colonize roots along with myriad other microbes, which are collectively called the “microbiome.” Many other microbial organisms, such as fungal endophytes and bacteria, can also confer stress tolerance in plants. The consideration of microbe-microbe interactions could be an exciting area for future research.
- The majority of these studies were conducted in controlled growth chamber or greenhouse conditions, while very few have been conducted in field conditions. We need more data regarding the variability or stability of AMF-conferred salinity tolerance before advocating for farm-level applications.
- From a mechanistic perspective, most papers measured the osmolyte concentration in AMF and non-AMF plants, but the underlying mechanisms of this response at the molecular level need further investigation.
- Very few studies have described the effects of sulfur, and they have made inconclusive findings (Evelin et al., 2019). We have yet to explore whether hormones, such as brassinosteroids, auxins, jasmonic acid, and salicylic acid, are involved in AMF-induced plant salinity tolerance, as some of these hormones have been found to improve plant tolerance to salinity (Pedranzani et al., 2003; Siddiqi and Husen, 2019).
- Under salinity stress, the plant lipid metabolism is altered, which is associated with alterations in membrane integrity, composition, and function (Parihar et al., 2015). Lipid peroxidation has been studied, but lipid metabolism in salt-stressed AMF-inoculated plants has received less attention.

Salinity stress inhibits plant growth and the accumulation of biomass by affecting photosynthesis, osmotic balance, enzymatic activities, and nutrient uptake. Mycorrhizal fungi consistently help plants to reduce the impact of salt stress by modulating their physiological processes. AMF-induced plant salinity stress tolerance has broad ecological and agricultural implications. In some regions, increased salinity tolerance can result in higher crop yields. Continued interest in AMF research to uncover the underlying mechanisms of plant-AMF interactions appears well-justified.

## Conclusion

Our current meta-analysis of 97 published peer reviews related to the effect of AMF on plant responses under salinity stress revealed that compared to non-inoculated plants, AMF plants had significantly higher shoot and root biomass regardless of salinity stress and that the effect was more prominent as salinity stress increased. Consistent with the previous findings of Chandrasekaran et al. (2014, 2016, 2019), Wang et al. (2019), and Pan et al. (2020), we report that AMF-mediated plant salinity tolerance was influenced by fungal genera, plant clades and plant photosynthetic pathways. In addition, we observed for the first time that significant phylogenetic signals exist in AMF-mediated plant salinity tolerance, i.e., closely related plant species had more similar responses to moderate salinity stress when inoculated with closely related (phylogenetic) AMF species. Under salinity stress, the growth of C3 plants was more strongly influenced by AMF inoculation than that of C4 plants, which corroborates the findings of another meta-analysis (Chandrasekaran et al., 2016). However, our analyses did not show any positive influence of AMF on the shoot biomass of C4 plants, which is somewhat contradictory to the findings of Chandrasekaran et al. (2016). Furthermore, we showed that the effect of AMF treatment in plants was more pronounced at longer durations of salinity stress (>2 weeks). Therefore, choosing the appropriate host plant and AMF species is important for using plant–AMF symbionts to improve salt-affected soil in practical applications. The inoculation of AMF consistently increases shoot K and decreases shoot Na, as demonstrated by several investigators (Augé et al., 2014; Chandrasekaran et al., 2014; Pan et al., 2020) and the outcomes of this meta-analysis. The involvement of CAT, SOD, and POD has been reported in previous meta-analyses (Chandrasekaran et al., 2014; Pan et al., 2020), but we also found that AMF-mediated plant salinity tolerance at low salinity was associated with higher SOD and POD activity, whereas at moderate salinity, more CAT and proline accumulation were also observed in addition to POD and SOD enzymes. AMF-mediated plant salinity tolerance has broad ecological and agricultural implications.

## Author Contributions

KD developed the concept, performed the database search, partially collected the data, analyzed and interpreted the results, and wrote the draft. MZ, MT, and MA extracted the data, produced the graphs, discussed the results, and revised the manuscript. SO revised the manuscript and supervised the project. All authors approved the final version of the manuscript.

## Conflict of Interest

The authors declare that the research was conducted in the absence of any commercial or financial relationships that could be construed as a potential conflict of interest.

## Supplementary Material

Supplementary Figure 1 Pie chart showing frequency (%) of occurrences of plant **(A)** and fungal **(B)** species in the whole metaanalysis dataset in current study.

Supplementary Table 1 List of papers used in metanalysis.

Supplementary Table 2 Metadata obtained and used for analyses in the current study.

Supplementary Table 3 Evaluating bias caused by non-independence of multiple data points per study.
