# Supplementary figures and images for: Plant Salinity Tolerance Conferred by Arbuscular Mycorrhizal Fungi and Associated Mechanisms: A Meta-Analysis
Source: Front Plant Sci. 2020 Dec 9;11:588550. doi: 10.3389/fpls.2020.588550 (PMC7755987; doi:10.3389/fpls.2020.588550)

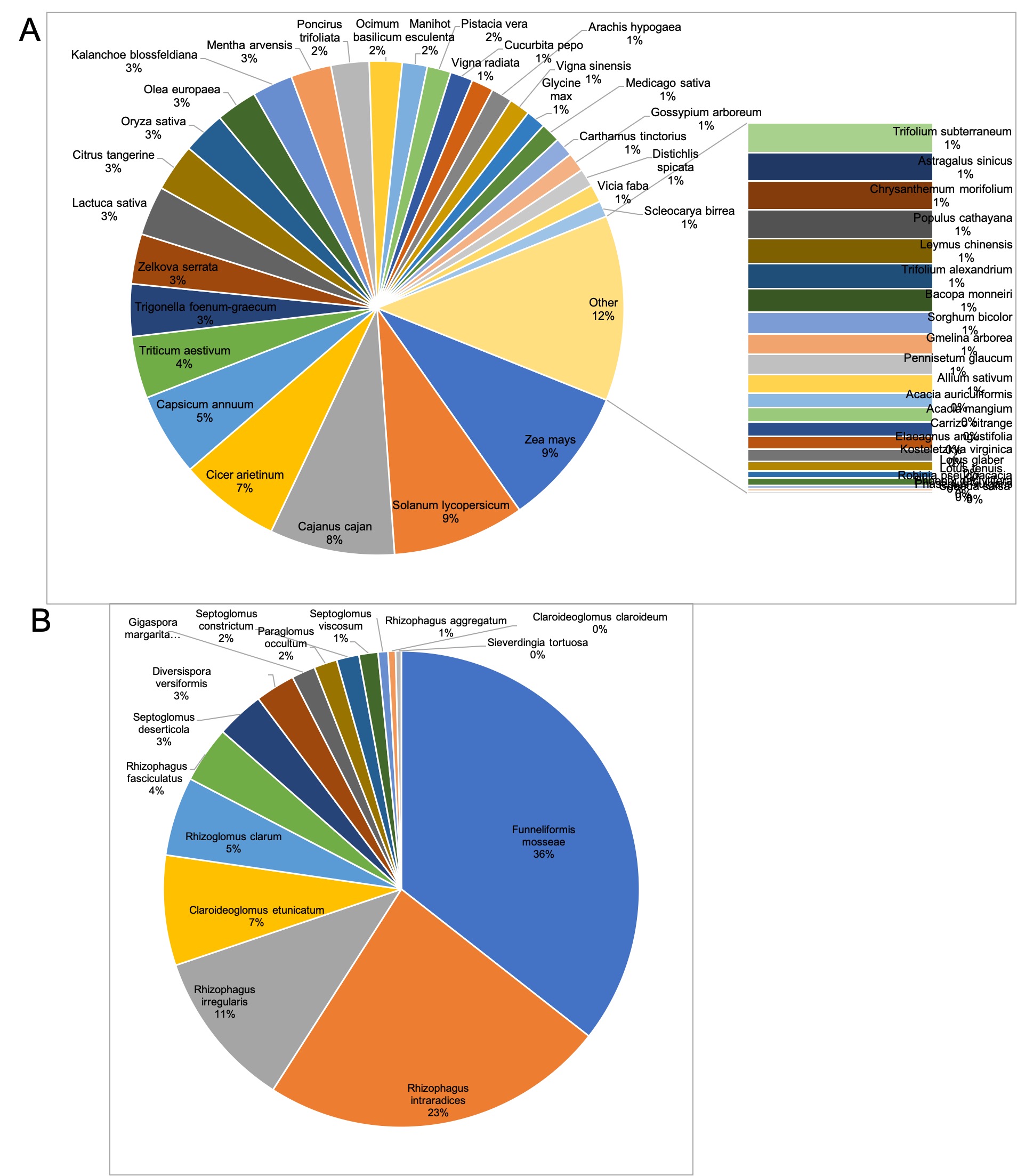

Supplement: Supplementary Figure 1 — Pie chart showing frequency (%) of occurrences of plant (A) and fungal (B) species in the whole metaanalysis dataset in current study. [file Image_1.JPEG]
