# Supplementary material for: Plant Salinity Tolerance Conferred by Arbuscular Mycorrhizal Fungi and Associated Mechanisms: A Meta-Analysis
Source: Front Plant Sci. 2020 Dec 9;11:588550. doi: 10.3389/fpls.2020.588550 (PMC7755987; doi:10.3389/fpls.2020.588550)
Supplement: Supplementary Table 1 — List of papers used in metanalysis. [file Table_1.DOCX]

**Supplementary Table 1: List of Papers used in metanalysis:**

1. Abbaspour, H. (2010). Investigation of the effects of vesicular arbuscular mycorrhiza on mineral nutrition and growth of Carthamus tinctorius under salt stress conditions. Russian journal of plant physiology 57, 526-531.
2. Abbaspour, H., Fallahyan, F., Fahimi, H., and Afshari, H. (2006). Response of Pistacia vera L. in salt tolerance to inoculation with arbuscular mycorrhizal fungi under salt stress. Acta horticulturae.
3. Abdel-Fattah, G. M., Ibrahim, A. H., Al-Amri, S. M., and Shoker, A. E. (2013). Synergistic effect of arbuscular mycorrhizal fungi and spermine on amelioration of salinity stress of wheat ('Triticum aestivum'L. cv. gimiza 9). Australian Journal of Crop Science 7, 1525.
4. Al-Karaki, G. N. (2000). Growth of mycorrhizal tomato and mineral acquisition under salt stress. Mycorrhiza 10, 51-54.
5. Al-Karaki, G. (2001). Salt stress response of salt-sensitive and tolerant durum wheat cultivars inoculated with mycorrhizal fungi. Acta Agronomica Hungarica 49, 25-34.
6. Al-Khaliel, A. (2010). Effect of salinity stress on mycorrhizal association and growth response of peanut infected by Glomus mosseae. Plant, Soil and Environment 56, 318-324.
7. Aroca, R., Porcel, R., and Ruiz‐Lozano, J. M. (2007). How does arbuscular mycorrhizal symbiosis regulate root hydraulic properties and plasma membrane aquaporins in Phaseolus vulgaris under drought, cold or salinity stresses? New Phytologist 173, 808-816.
8. Aroca, R., Ruiz-Lozano, J. M., Zamarreño, Á. M., Paz, J. A., García-Mina, J. M., Pozo, M. J., and López-Ráez, J. A. (2013). Arbuscular mycorrhizal symbiosis influences strigolactone production under salinity and alleviates salt stress in lettuce plants. Journal of plant physiology 170, 47-55.
9. Asghari, H. (2008). Vesicular-arbuscular (VA) mycorrhizae improve salinity tolerance in pre-inoculation subterranean clover (Trifolium subterraneum) seedlings. International Journal of Plant Production 2, 243-256.
10. Asrar, A. W. A., Abdel-Fattah, G. M., Elhindi, K. M., and Abdel-Salam, E. M. (2014). The impact of arbuscular mychorrhizal fungi in improving growth, flower yield and tolerance of kalanchoe (Kalanchoe blossfeldiana Poelin) plants grown in NaCl-stress conditions. Journal of Food, Agriculture and Environment 12, 105-112.
11. Augé, R. M., Toler, H. D., Sams, C. E., and Nasim, G. (2008). Hydraulic conductance and water potential gradients in squash leaves showing mycorrhiza-induced increases in stomatal conductance. Mycorrhiza 18, 115-121.
12. Bach Allen, E., and Cunningham, G. L. (1983). Effects of vesicular–arbuscular mycorrhizae on Distichlis spicata under three salinity levels. New Phytologist 93, 227-236.
13. Beltrano, J., Ruscitti, M., Arango, M., and Ronco, M. (2013). Effects of arbuscular mycorrhiza inoculation on plant growth, biological and physiological parameters and mineral nutrition in pepper grown under different salinity and p levels. Journal of soil science and plant nutrition 13, 123-141.
14. Bharti, A., and Garg, N. (2019). SA and AM symbiosis modulate antioxidant defense mechanisms and asada pathway in chickpea genotypes under salt stress. Ecotoxicology and Environmental Safety 178, 66-78.
15. Bharti, N., Baghel, S., Barnawal, D., Yadav, A., and Kalra, A. (2013). The greater effectiveness of Glomus mosseae and Glomus intraradices in improving productivity, oil content and tolerance of salt-stressed menthol mint (*Mentha arvensis*). Journal of the Science of Food and Agriculture 93, 2154-2161.
16. Bharti, N., Barnawal, D., Wasnik, K., Tewari, S. K., and Kalra, A. (2016). Co-inoculation of Dietzia natronolimnaea and Glomus intraradices with vermicompost positively influences Ocimum basilicum growth and resident microbial community structure in salt affected low fertility soils. Applied Soil Ecology 100, 211-225.
17. Borde, M., Dudhane, M., and Jite, P. (2011). Growth photosynthetic activity and antioxidant responses of mycorrhizal and non-mycorrhizal bajra (*Pennisetum glaucum*) crop under salinity stress condition. Crop Protection 30, 265-271.
18. Borde, M., Dudhane, M., and Jite, P. K. (2010). AM fungi influences the photosynthetic activity, growth and antioxidant enzymes in *Allium sativum* L. under salinity condition. Notulae Scientia Biologicae 2, 64-71.
19. Campanelli, A., Ruta, C., De Mastro, G., and Morone-Fortunato, I. (2013). The role of arbuscular mycorrhizal fungi in alleviating salt stress in Medicago sativa L. var. icon. Symbiosis 59, 65-76.
20. Carretero, C. L., Cantos, M., Garcia, J. L., Azcón, R., and Troncoso, A. (2008). Arbuscular-mycorrhizal contributes to alleviation of salt damage in cassava clones. Journal of Plant Nutrition 31, 959-971.
21. Chang, W., Sui, X., Fan, X.-X., Jia, T.-T., and Song, F.-Q. (2018). Arbuscular mycorrhizal symbiosis modulates antioxidant response and ion distribution in salt-stressed Elaeagnus angustifolia seedlings. Frontiers in microbiology 9, 652.
22. Chen, J., Zhang, H., Zhang, X., and Tang, M. (2017). Arbuscular mycorrhizal symbiosis alleviates salt stress in black locust through improved photosynthesis, water status, and K+/Na+ homeostasis. Frontiers in plant science 8, 1739.
23. Datta, P., and Kulkarni, M. (2014). Influence of two "AM" fungi in improvement of mineral profile in Arachis hypogaea L. under salinity stress. Legume Research 37, 321-328.
24. Diouf, D., Duponnois, R., Ba, A. T., Neyra, M., and Lesueur, D. (2005). Symbiosis of Acacia auriculiformis and Acacia mangium with mycorrhizal fungi and Bradyrhizobium spp. improves salt tolerance in greenhouse conditions. Functional plant biology 32, 1143-1152.
25. Dudhane, M. P., Borde, M. Y., and Jite, P. K. (2011). Effect of arbuscular mycorrhizal fungi on growth and antioxidant activity in Gmelina arborea Roxb. under salt stress condition. Notulae Scientia Biologicae 3, 71-78.
26. Duke, E., Johnson, C., and Koch, K. (1986). Accumulation of phosphorus, dry matter and betaine during NaCl stress of split‐root citrus seedlings colonized with vesicular‐arbuscular mycorrhizal fungi on zero, one or two halves. New phytologist 104, 583-590.
27. Echeverria, M., Scambato, A. A., Sannazzaro, A. I., Maiale, S., Ruiz, O. A., and Menéndez, A. B. (2008). Phenotypic plasticity with respect to salt stress response by Lotus glaber: the role of its AM fungal and rhizobial symbionts. Mycorrhiza 18, 317-329.
28. Echeverria, M., Sannazzaro, A. I., Ruiz, O. A., and Menéndez, A. B. (2013). Modulatory effects of Mesorhizobium tianshanense and Glomus intraradices on plant proline and polyamine levels during early plant response of Lotus tenuis to salinity. Plant and soil 364, 69-79.
29. Elhindi, K. M., El-Din, A. S., and Elgorban, A. M. (2017). The impact of arbuscular mycorrhizal fungi in mitigating salt-induced adverse effects in sweet basil (Ocimum basilicum L.). Saudi journal of biological sciences 24, 170-179.
30. Estrada, B., Barea, J. M., Aroca, R., and Ruiz-Lozano, J. M. (2013c). A native Glomus intraradices strain from a Mediterranean saline area exhibits salt tolerance and enhanced symbiotic efficiency with maize plants under salt stress conditions. Plant and Soil 366, 333-349.
31. Estrada, B., Aroca, R., Barea, J. M., and Ruiz-Lozano, J. M. (2013a). Native arbuscular mycorrhizal fungi isolated from a saline habitat improved maize antioxidant systems and plant tolerance to salinity. Plant science 201, 42-51.
32. Evelin, H., and Kapoor, R. (2014). Arbuscular mycorrhizal symbiosis modulates antioxidant response in salt-stressed Trigonella foenum-graecum plants. Mycorrhiza 24, 197-208.
33. Evelin, H., Giri, B., and Kapoor, R. (2012). Contribution of Glomus intraradices inoculation to nutrient acquisition and mitigation of ionic imbalance in NaCl-stressed Trigonella foenum-graecum. Mycorrhiza 22, 203-217.
34. Evelin, H., Giri, B., and Kapoor, R. (2013). Ultrastructural evidence for AMF mediated salt stress mitigation in Trigonella foenum-graecum. Mycorrhiza 23, 71-86.
35. Garg, N., and Baher, N. (2013). Role of arbuscular mycorrhizal symbiosis in proline biosynthesis and metabolism of Cicer arietinum L.(chickpea) genotypes under salt stress. Journal of plant growth regulation 32, 767-778.
36. Garg, N., and Bhandari, P. (2016a). Interactive effects of silicon and arbuscular mycorrhiza in modulating ascorbate-glutathione cycle and antioxidant scavenging capacity in differentially salt-tolerant Cicer arietinum L. genotypes subjected to long-term salinity. Protoplasma 253, 1325-1345.
37. Garg, N., and Bhandari, P. (2016b). Silicon nutrition and mycorrhizal inoculations improve growth, nutrient status, K+/Na+ ratio and yield of Cicer arietinum L. genotypes under salinity stress. Plant growth regulation 78, 371-387.
38. Garg, N., and Bharti, A. (2018). Salicylic acid improves arbuscular mycorrhizal symbiosis, and chickpea growth and yield by modulating carbohydrate metabolism under salt stress. Mycorrhiza 28, 727-746.
39. Garg, N., and Chandel, S. (2011). The effects of salinity on nitrogen fixation and trehalose metabolism in mycorrhizal Cajanus cajan (L.) Millsp. plants. Journal of plant growth regulation 30, 490-503.
40. Garg, N., and Chandel, S. (2012). Role of arbuscular mycorrhizal (AM) fungi on growth, cadmium uptake, osmolyte, and phytochelatin synthesis in Cajanus cajan (L.) Millsp. under NaCl and Cd stresses. Journal of plant growth regulation 31, 292-308.
41. Garg, N., and Manchanda, G. (2009). Role of arbuscular mycorrhizae in the alleviation of ionic, osmotic and oxidative stresses induced by salinity in Cajanus cajan (L.) Millsp.(pigeonpea). Journal of Agronomy and Crop Science 195, 110-123.
42. Garg, N., and Pandey, R. (2016). High effectiveness of exotic arbuscular mycorrhizal fungi is reflected in improved rhizobial symbiosis and trehalose turnover in Cajanus cajan genotypes grown under salinity stress. Fungal Ecology 21, 57-67.
43. Ghorbanli, M., Ebrahimzadeh, H., and Sharifi, M. (2004). Effects of NaCl and mycorrhizal fungi on antioxidative enzymes in soybean. Biologia Plantarum 48, 575-581.
44. Hajiboland, R., Aliasgharzadeh, N., Laiegh, S. F., and Poschenrieder, C. (2010). Colonization with arbuscular mycorrhizal fungi improves salinity tolerance of tomato (Solanum lycopersicum L.) plants. Plant and Soil 331, 313-327.
45. He, Z., and Huang, Z. (2013). Expression analysis of LeNHX1 gene in mycorrhizal tomato under salt stress. Journal of Microbiology 51, 100-104.
46. He, Z., He, C., Zhang, Z., Zou, Z., and Wang, H. (2007). Changes of antioxidative enzymes and cell membrane osmosis in tomato colonized by arbuscular mycorrhizae under NaCl stress. Colloids and Surfaces B: Biointerfaces 59, 128-133.
47. Hirrel, M. C., and Gerdemann, J. (1980). Improved growth of onion and bell pepper in saline soils by two vesicular-arbuscular mycorrhizal fungi 1. Soil Science Society of America Journal 44, 654-655.
48. Huang, J.-C., Lai, W.-A., Singh, S., Hameed, A., and Young, C.-C. (2013). Response of mycorrhizal hybrid tomato cultivars under saline stress. Journal of soil science and plant nutrition 13, 469-484.
49. Huang, Z., He, C.-X., He, Z.-Q., Zou, Z.-R., and Zhang, Z.-B. (2010). The effects of arbuscular mycorrhizal fungi on reactive oxyradical scavenging system of tomato under salt tolerance. Agricultural Sciences in China 9, 1150-1159.
50. Jahromi, F., Aroca, R., Porcel, R., and Ruiz-Lozano, J. M. (2008). Influence of salinity on the in vitro development of Glomus intraradices and on the in vivo physiological and molecular responses of mycorrhizal lettuce plants. Microbial Ecology 55, 45.
51. Kaya, C., Ashraf, M., Sonmez, O., Aydemir, S., Tuna, A. L., and Cullu, M. A. (2009). The influence of arbuscular mycorrhizal colonisation on key growth parameters and fruit yield of pepper plants grown at high salinity. Scientia Horticulturae 121, 1-6.
52. Khaliel, A., Shine, K., and Vijayakumar, K. (2011). Salt tolerance and mycorrhization of Bacopa monneiri grown under sodium chloride saline conditions. African Journal of Microbiology Research 5, 2034-2040.
53. Khalloufi, M., Martínez-Andújar, C., Lachaâl, M., Karray-Bouraoui, N., Pérez-Alfocea, F., and Albacete, A. (2017). The interaction between foliar GA3 application and arbuscular mycorrhizal fungi inoculation improves growth in salinized tomato (Solanum lycopersicum L.) plants by modifying the hormonal balance. Journal of plant physiology 214, 134-144.
54. Kohler, J., Caravaca, F., and Roldan, A. (2010). An AM fungus and a PGPR intensify the adverse effects of salinity on the stability of rhizosphere soil aggregates of Lactuca sativa. Soil Biology & Biochemistry 42, 429-434.
55. Kohler, J., Hernandez, J. A., Caravaca, F., and Roldan, A. (2009). Induction of antioxidant enzymes is involved in the greater effectiveness of a PGPR versus AM fungi with respect to increasing the tolerance of lettuce to severe salt stress. Environmental and Experimental Botany 65, 245-252.
56. Latef, A. A. H. A., and Chaoxing, H. (2014). Does inoculation with Glomus mosseae improve salt tolerance in pepper plants? Journal of Plant Growth Regulation 33, 644-653.
57. Latef, A. A. H. A., and Chaoxing, H. (2011). Effect of arbuscular mycorrhizal fungi on growth, mineral nutrition, antioxidant enzymes activity and fruit yield of tomato grown under salinity stress. Scientia Horticulturae 127, 228-233.
58. Lin, J., Wang, Y., Sun, S., Mu, C., and Yan, X. (2017). Effects of arbuscular mycorrhizal fungi on the growth, photosynthesis and photosynthetic pigments of Leymus chinensis seedlings under salt-alkali stress and nitrogen deposition. Science of the Total Environment 576, 234-241.
59. Liu, H., Wang, Y., Chen, H., and Tang, M. (2017). Influence of Rhizoglomus irregulare on nutraceutical quality and regeneration of Lycium barbarum leaves under salt stress. Canadian Journal of Microbiology 63, 365-374.
60. Muok, B. O. (2006). Effect of arbuscular mycorrhizal fungi on tree growth and nutrient uptake of Sclerocarya birrea under water stress, salt stress and flooding. 園芸学会雑誌 75, 26-31.
61. Pandey, R., and Garg, N. (2017). High effectiveness of Rhizophagus irregularis is linked to superior modulation of antioxidant defence mechanisms in Cajanus cajan (L.) Millsp. genotypes grown under salinity stress. Mycorrhiza 27, 669-682.
62. Peng, J., Li, Y., Shi, P., Chen, X., Lin, H., and Zhao, B. (2011). The differential behavior of arbuscular mycorrhizal fungi in interaction with Astragalus sinicus L. under salt stress. Mycorrhiza 21, 27-33.
63. Porcel, R., Redondo-Gómez, S., Mateos-Naranjo, E., Aroca, R., Garcia, R., and Ruiz-Lozano, J. M. (2015). Arbuscular mycorrhizal symbiosis ameliorates the optimum quantum yield of photosystem II and reduces non-photochemical quenching in rice plants subjected to salt stress. Journal of plant physiology 185, 75-83.
64. Porcel, R., Aroca, R., Azcon, R., and Ruiz-Lozano, J. M. (2016). Regulation of cation transporter genes by the arbuscular mycorrhizal symbiosis in rice plants subjected to salinity suggests improved salt tolerance due to reduced Na+ root-to-shoot distribution. Mycorrhiza 26, 673-684.
65. Porras-Soriano, A., Soriano-Martín, M. L., Porras-Piedra, A., and Azcón, R. (2009). Arbuscular mycorrhizal fungi increased growth, nutrient uptake and tolerance to salinity in olive trees under nursery conditions. Journal of plant physiology 166, 1350-1359.
66. Rabie, G. (2005). Influence of arbuscular mycorrhizal fungi and kinetin on the response of mungbean plants to irrigation with seawater. Mycorrhiza 15, 225-230.
67. Rabie, G., and Almadini, A. (2005). Role of bioinoculants in development of salt-tolerance of Vicia faba plants under salinity stress. African Journal of Biotechnology 4, 210-222.
68. Rabie, G., Aboul-Nasr, M., and Al-Humiany, A. (2005). Increased salinity tolerance of cowpea plants by dual inoculation of an arbuscular mycorrhizal fungus Glomus clarum and a nitrogen-fixer Azospirillum brasilense. Mycobiology 33, 51-60.
69. Rinaldelli, E., and Mancuso, S. (1996). Response of young mycorrhizal and non-mycorrhizal plants of olive tree (Olea europaea L.) to saline conditions. I. Short-term electrophysiological and long-term vegetative salt effects. Advances in Horticultural Science, 126-134.
70. Selvakumar, G., and Thamizhiniyan, P. (2011). The effect of the arbuscular mycorrhizal (AM) fungus Glomus intraradices on the growth and yield of chilli (Capsicum annuum L.) under salinity stress. World Appl Sci J 14, 1209-1214.
71. Shabbir, G., Dakheel, A. J., and Al-Naqbi, M. R. S. (2010). The effect of arbuscular mycorrhize (AM) fungi on the establishment of date palm (phoenix dactylifera l.) under saline conditions in the UAE. In "Acta Horticulturae", Vol. 882, pp. 303-314.
72. Shamshiri, M., and Fattahi, M. (2016). Effects of arbuscular mycorrhizal fungi on photosystem II activity of three Pistachio rootstocks under salt stress as probed by the OJIP-test. Russian journal of plant physiology 63, 101-110.
73. Shekoofeh, E., Sepideh, H., and Roya, R. (2012). Role of mycorrhizal fungi and salicylic acid in salinity tolerance of Ocimum basilicum resistance to salinity. African Journal of Biotechnology 11, 2223-2235.
74. Sheng, M., Tang, M., Chen, H., Yang, B., Zhang, F., and Huang, Y. (2008). Influence of arbuscular mycorrhizae on photosynthesis and water status of maize plants under salt stress. Mycorrhiza 18, 287-296.
75. Sheng, M., Tang, M., Zhang, F., and Huang, Y. (2011). Influence of arbuscular mycorrhiza on organic solutes in maize leaves under salt stress. Mycorrhiza 21, 423-430.
76. Shokri, S., and Maadi, B. (2009). Effects of arbuscular mycorrhizal fungus on the mineral nutrition and yield of Trifolium alexandrinum plants under salinity stress. J Agron 8, 79-83.
77. Tian, C., Feng, G., Li, X., and Zhang, F. (2004). Different effects of arbuscular mycorrhizal fungal isolates from saline or non-saline soil on salinity tolerance of plants. Applied Soil Ecology 26, 143-148.
78. Tofighi, C., Khavari-Nejad, R. A., Najafi, F., Razavi, K., and Rejali, F. (2017). Responses of wheat plants to interactions of 24-epibrassinolide and Glomus mosseae in saline condition. Physiology and Molecular Biology of Plants 23, 557-564.
79. Turkmen, O., Sensoy, S., Demir, S., and Erdinc, C. (2008). Effects of two different AMF species on growth and nutrient content of pepper seedlings grown under moderate salt stress. African Journal of Biotechnology 7, 392-396.
80. Wang, Y., Wang, M., Li, Y., Wu, A., and Huang, J. (2018). Effects of arbuscular mycorrhizal fungi on growth and nitrogen uptake of Chrysanthemum morifolium under salt stress. PloS one 13, e0196408.
81. Wang, J., Fu, Z., Ren, Q., Zhu, L., Lin, J., Zhang, J., Cheng, X., Ma, J., and Yue, J. (2019). Effects of Arbuscular Mycorrhizal Fungi on Growth, Photosynthesis, and Nutrient Uptake of Zelkova serrata (Thunb.) Makino Seedlings under Salt Stress. Forests 10, 186.
82. Wu, Q.-S., and Zou, Y.-N. (2009). Arbuscular mycorrhizal symbiosis improves growth and root nutrient status of citrus subjected to salt stress. ScienceAsia 35, 388-391.
83. Wu, Qiang-Sheng; Zou, Ying-Ning, 2009 Arbuscular mycorrhizal symbiosis improves growth and root nutrient status of citrus subjected to salt stress
84. Wu, Q.-S., Zou, Y.-N., and He, X.-H. (2010a). Contributions of arbuscular mycorrhizal fungi to growth, photosynthesis, root morphology and ionic balance of citrus seedlings under salt stress. Acta physiologiae plantarum 32, 297-304.
85. Wu, Q. S., Zou, Y. N., Liu, W., Ye, X., Zai, H., and Zhao, L. (2010b). Alleviation of salt stress in citrus seedlings inoculated with mycorrhiza: changes in leaf antioxidant defense systems. Plant, Soil and Environment 56, 470-475.
86. Wu, N., Li, Z., Liu, H., and Tang, M. (2015). Influence of arbuscular mycorrhiza on photosynthesis and water status of Populus cathayana Rehder males and females under salt stress. Acta physiologiae plantarum 37, 183.
87. Wu, Q.-S., Zou, Y.-N., and He, X.-H. (2013). Mycorrhizal symbiosis enhances tolerance to NaCl stress through selective absorption but not selective transport of K+ over Na+ in trifoliate orange. Scientia Horticulturae 160, 366-374.
88. Xu, H., Lu, Y., and Tong, S. (2018). Effects of arbuscular mycorrhizal fungi on photosynthesis and chlorophyll fluorescence of maize seedlings under salt stress. Emirates Journal of Food and Agriculture, 199-204.
89. Yamato, M., Ikeda, S., and Iwase, K. (2008). Community of arbuscular mycorrhizal fungi in a coastal vegetation on Okinawa island and effect of the isolated fungi on growth of sorghum under salt-treated conditions. Mycorrhiza 18, 241-249.
90. Younesi, O., Moradi, A., and Namdari, A. (2013). Influence of arbuscular mycorrhiza on osmotic adjustment compounds and antioxidant enzyme activity in nodules of salt-stressed soybean (Glycine max). Acta Agriculturae Slovenica 101, 219.
91. Zhang, H. S., Qin, F. F., Qin, P., and Pan, S. M. (2014). Evidence that arbuscular mycorrhizal and phosphate-solubilizing fungi alleviate NaCl stress in the halophyte Kosteletzkya virginica: nutrient uptake and ion distribution within root tissues. Mycorrhiza 24, 383-395.
92. He, Z., Tang, H., Li, H., He, C., Zhang, Z., and Wang, H. (2010). Arbuscular mycorrhizal alleviated ion toxicity, oxidative damage and enhanced osmotic adjustment in tomato subjected to NaCl stress. American-Eurasian Journal of Agricultural and Environmental Science 7, 676-683.
93. Zhu, X., Song, F., Liu, S., and Liu, F. (2016). Role of arbuscular mycorrhiza in alleviating salinity stress in wheat (Triticum aestivum L.) grown under ambient and elevated CO2. Journal of Agronomy and Crop Science 202, 486-496.
94. Zhu, X., Song, F., Liu, S., Liu, F., and Li, X. (2018). Arbuscular mycorrhiza enhances nutrient accumulation in wheat exposed to elevated CO2 and soil salinity. Journal of Plant Nutrition and Soil Science 181, 836-846.
95. Zou, Y.-N., and Wu, Q.-S. (2011). Sodium chloride stress induced changes in leaf osmotic adjustment of trifoliate orange (Poncirus trifoliata) seedlings inoculated with mycorrhizal fungi. Notulae Botanicae Horti Agrobotanici Cluj-Napoca 39, 64-69.
96. Zou, Y.-N., Liang, Y.-C., and Wu, Q.-S. (2013). Mycorrhizal and non-mycorrhizal responses to salt stress in trifoliate orange: plant growth, root architecture and soluble sugar accumulation. Int J Agric Biol 15, 565-569.
97. Zuccarini, P., and Okurowska, P. (2008). Effects of mycorrhizal colonization and fertilization on growth and photosynthesis of sweet basil under salt stress. Journal of Plant Nutrition 31, 497-513.
