# Supplementary material for: Plant Salinity Tolerance Conferred by Arbuscular Mycorrhizal Fungi and Associated Mechanisms: A Meta-Analysis
Source: Front Plant Sci. 2020 Dec 9;11:588550. doi: 10.3389/fpls.2020.588550 (PMC7755987; doi:10.3389/fpls.2020.588550)
Supplement: Supplementary Table 3 — Evaluating bias caused by non-independence of multiple data points per study. [file Table_3.DOCX]

**ST3. Evaluating bias caused by non-independence of multiple data points per study.** Heterogeneity (Q_t_), sample size, effect sizes (SMD) and 95% CI for different fitness components when considering all data points (full data), only one data point per article (reduced) and percent changes of SMD and significance tests (p=value) using Welch Two Sample t-test of effect sizes of all studies grouped under full and reduced data points.

|  | Qt | N | SMD | L95%CI | U95%CI | %Change of SMD | p-value |
| --- | --- | --- | --- | --- | --- | --- | --- |
| Shoot Dry Biomass |  |  |  |  |  |  |  |
| Non-Saline (full) | 123.11 | 120 | 0.607 | 0.491 | 0.723 | -10.880 | 0.386 |
| Non-Saline (reduced) | 59.22 | 61 | 0.673 | 0.505 | 0.840 |  |  |
| Low Saline (full) | 190.32 | 124 | 0.799 | 0.644 | 0.954 | -3.217 | 0.911 |
| Low Saline (reduced) | 68.78 | 50 | 0.825 | 0.602 | 1.047 |  |  |
| Moderate Saline (full) | 141.96 | 108 | 0.807 | 0.663 | 0.951 | -5.627 | 0.662 |
| Moderate Saline (reduced) | 81.64 | 58 | 0.852 | 0.638 | 1.066 |  |  |
| High Saline (full) | 20.99 | 15 | 0.952 | 0.568 | 1.337 | 18.354 | 0.615 |
| High Saline (reduced) | 13.93 | 9 | 0.778 | 0.245 | 1.310 |  |  |
| Root Dry Biomass |  |  |  |  |  |  |  |
| Non-Saline (full) | 212.64 | 97 | 0.585 | 0.395 | 0.776 | -15.140 | 0.547 |
| Non-Saline (reduced) | 118.88 | 59 | 0.674 | 0.422 | 0.926 |  |  |
| Low Saline (full) | 160.44 | 95 | 0.705 | 0.507 | 0.903 | -12.537 | 0.639 |
| Low Saline (reduced) | 85.08 | 46 | 0.794 | 0.473 | 1.114 |  |  |
| Moderate Saline (full) | 106.28 | 86 | 0.817 | 0.659 | 0.976 | 6.937 | 0.856 |
| Moderate Saline (reduced) | 66.56 | 44 | 0.761 | 0.499 | 1.022 |  |  |
| High Saline (full) | 14.28 | 9 | 1.235 | 0.556 | 1.914 | -21.751 | 0.705 |
| High Saline (reduced) | 8.25 | 6 | 1.504 | 0.253 | 2.754 |  |  |
| Plant Height |  |  |  |  |  |  |  |
| Non-Saline (full) | 6.03 | 25 | 0.6307 | 0.4913 | 0.77 | -2.045 | 0.8634 |
| Non-Saline (reduced) | 5.16 | 22 | 0.6436 | 0.4981 | 0.789 |  |  |
| Low Saline (full) | 9.60 | 23 | 0.7591 | 0.5673 | 0.9509 | 1.107 | 0.9103 |
| Low Saline (reduced) | 7.22 | 13 | 0.7507 | 0.4384 | 1.063 |  |  |
| Moderate Saline (full) | 10.28 | 23 | 0.5481 | 0.3427 | 0.7536 | -17.752 | 0.5056 |
| Moderate Saline (reduced) | 7.72 | 16 | 0.6454 | 0.3729 | 0.9179 |  |  |
| High Saline (full) | 1.06 | 8 | 0.7338 | 0.4947 | 0.9729 | 7.332 | 0.8167 |
| High Saline (reduced) | 0.92 | 5 | 0.68 | 0.2211 | 1.132 |  |  |
